# Classifying Breast Cancer Molecular Subtypes by Using Deep Clustering Approach

**DOI:** 10.3389/fgene.2020.553587

**Published:** 2020-11-25

**Authors:** Narjes Rohani, Changiz Eslahchi

**Affiliations:** ^1^Department of Computer and Data Sciences, Faculty of Mathematics, Shahid Beheshti University, Tehran, Iran; ^2^School of Biological Sciences, Institute for Research in Fundamental Sciences (IPM), Tehran, Iran

**Keywords:** cancer molecular subtypes, breast cancer, machine learning, somatic mutations, clustering, tumor classification

## Abstract

Cancer is a complex disease with a high rate of mortality. The characteristics of tumor masses are very heterogeneous; thus, the appropriate classification of tumors is a critical point in the effective treatment. A high level of heterogeneity has also been observed in breast cancer. Therefore, detecting the molecular subtypes of this disease is an essential issue for medicine that could be facilitated using bioinformatics. This study aims to discover the molecular subtypes of breast cancer using somatic mutation profiles of tumors. Nonetheless, the somatic mutation profiles are very sparse. Therefore, a network propagation method is used in the gene interaction network to make the mutation profiles dense. Afterward, the deep embedded clustering (DEC) method is used to classify the breast tumors into four subtypes. In the next step, gene signature of each subtype is obtained using Fisher's exact test. Besides the enrichment of gene signatures in numerous biological databases, clinical and molecular analyses verify that the proposed method using mutation profiles can efficiently detect the molecular subtypes of breast cancer. Finally, a supervised classifier is trained based on the discovered subtypes to predict the molecular subtype of a new patient. The code and material of the method are available at: https://github.com/nrohani/MolecularSubtypes.

## 1. Introduction

Breast cancer is a heterogeneous disease at the molecular and clinical levels; thus, the effectiveness of a treatment is hugely different based on the tumor characteristics. This heterogeneity is a challenge for tumor classification to reach an appropriate clinical outcome. To solve this problem, many researchers have developed numerous methods to classify tumor masses, such as histopathological classification based on the morphological characteristics or immunohistochemical (IHC) markers such as estrogen receptor (ER), progesterone receptor (PR), and human epidermal growth factor receptor 2 (HER2) (Elston, 1999; Perou et al., 2000; Sørlie et al., 2001; Hu et al., 2006; Hofree et al., 2013; Ali et al., 2014; List et al., 2014). Moreover, Sorlie et al. have used hierarchical clustering on the gene expression data that led to the identification of significant breast cancer subtypes (Perou et al., 2000). The high cost of gene expression analysis for many genes was a significant obstacle in applying this method. To overcome this issue, the researchers have reduced the gene list to a relevant gene signature for breast cancer subtypes detection. Parker et al. (2009) have presented biomarker genes that can efficiently detect molecular subtypes. These genes could be an excellent alternative to whole transcriptome microarray analysis. The subtypes found by these genes are known as *PAM*50 subtypes. Diversity of gene expression data in the subtypes is an indicator for the clinical prognosis of the patients, such as survival outcome (Sørlie et al., 2003).

In some studies, the microarray-based breast cancer classification has been considered as the gold standard (Peppercorn et al., 2007). However, the microarray-based methods cannot classify tumors consistently, due to the dynamic nature of gene expression data (Pusztai et al., 2006; Gusterson, 2009; Weigelt et al., 2010).

Some studies have recently identified cancer subtypes based on somatic mutation profiles of tumors (Vural et al., 2016; Zhang et al., 2018b; Kuijjer et al., 2018). Somatic mutations are more stable and have critical functions in cancer development and progression (Vural et al., 2016; Kuijjer et al., 2018). Moreover, investigating somatic mutation profiles can aid in cancer diagnosis and treatment due to the vast number of clinical guidelines based on single gene mutation (Kuijjer et al., 2018). Therefore, the classification of cancers based on the mutation profiles can help identify subtypes of patients and their treatments (Pusztai et al., 2006; Gusterson, 2009; Weigelt et al., 2010; Kuijjer et al., 2018). On the other side, with the development of new sequencing technologies, genome sequencing has become an appropriate tool for diagnostic purposes. Therefore, tumor classification based on somatic mutation profiles and application of the results in the clinical decisions can be crucial in the personalized medicine (Kuijjer et al., 2018).

Some studies have merged different kinds of the molecular data for breast cancer classification. Curtis et al. (2012) have developed a method to classify breast cancer by integrating genome and transcriptome data of 2,000 breast cancer patients. Based on the impact of somatic copy number alterations (CNAs) on the transcriptome, they have introduced new subtypes for breast cancer. Furthermore, Ali et al. (2014) have classified breast cancer into ten subtypes based on the combination of CNAs and gene expression data. In another study, List et al. (2014) have proposed a machine learning-based method that merges the gene expression and DNA methylation data for breast cancer classification. In a novel study, Hofree et al. (2013) have proposed a network stratification algorithm to classify tumors by fusing somatic mutation profiles with gene interaction network and have identified four subtypes for breast cancer. As somatic mutations are often sparse, it is sometimes challenging to predict cancer subtypes using somatic mutations. Therefore, previous studies have used other molecular information beside the somatic mutation data to detect cancer subtypes (Hofree et al., 2013).

In the most previous works, conventional clustering methods have been used to classify tumors; however, numerous innovative clustering methods have been proposed recently with various capabilities, which may help identify cancer subtypes. Moreover, the number of clusters typically has been determined using the silhouette criterion, which may lead to biologically meaningless clusters. In addition to the mentioned issues, the discovered clusters using somatic mutations are not analyzed extensively in previous works. In this study, the novel subtypes are presented using analysis of the somatic mutations and CNAs data from 861 breast tumors in the cancer genome atlas (TCGA) database (The International Cancer Genome Consortium, 2010). We used the network propagation method for smoothing somatic mutation profiles besides the gene interaction network; then, we used deep embedded clustering (DEC) (Xie et al., 2016) to find new breast cancer subtypes. Moreover, we used novel metrics such as AUMF (Maddi et al., 2019) and MMR (Brohee and Van Helden, 2006) for finding the best number of clusters. Afterward, the biological features of discovered subtypes were analyzed. Finally, a supervised model was trained to predict the breast cancer subtype of new patients. Also, the random forest (RF) was used to find the most important genes for classification.

## 2. Materials and Methods

### 2.1. Extracting and Smoothing Data

We used somatic mutation profiles collected by Zhang et al. (2018b). They have obtained somatic mutation data of 861 breast tumors from TCGA. A gene is recognized altered if at least one of the following conditions satisfies:

- It has a non-silent somatic mutation.
- It is a well-defined oncogene or tumor suppressor.
- It happens within a CNA.

The somatic mutation profiles are sparse, that is, in each tumor, the number of mutated genes is relatively small compared to the total number of genes (Hofree et al., 2013; Zhang et al., 2018a). In most machine learning techniques, sparse data cannot train the model well (Zhang et al., 2018a), so data need to be smoothed. One of the most effective solutions for smoothing data is the network propagation (Hofree et al., 2013). By combining somatic mutation profiles and gene interaction networks, we can obtain profiles that are not sparse. Here, the protein–protein interaction (PPI) information in the STRING database (Szklarczyk et al., 2016) was used to create a gene interaction network. For this purpose, the *Homosapiens* PPI network was obtained from the STRING database. Then, the gene interaction network was created from the PPI network by mapping proteins to genes. The mutation profile of each tumor was integrated with the gene interaction network. In fact, the entire vertices of the network were labeled based on the mutation profile of each tumor. If a gene is mutated, the corresponding vertex is labeled one, and zero otherwise.

Then, in the network propagation process, a random walk with restart was applied on the networks as Equation (1).

(1) $$\begin{array}{l} D_{i+1}=\alpha D_{i}A+\left(1-\alpha \right)D_{0},\;i=0,1,2,... \end{array}$$

The adjustment parameter α controls the amount of distance that a mutation can be propagated in the network. The optimal value of α varies for each network (in this study, it is subjectively set to 0.4). The network propagation process iterates until *D*_*i*+1_ is converged (i.e., $\|D_{i+1}-D_{i}\|<1\times 10^{-6}$). *D*_0_ is the original profile of tumor mutations, which is a *k* × *n* matrix (*k* is the number of tumors and *n* is the number of genes). *D*_*i*_ is the modified profile of mutations in the *i*th iteration. Matrix *A* is computed by *A* = *H* × *D*, where *H* = [*h*_*ij*_] is the adjacent matrix of the network and *D* = [*d*_*ij*_] is a diagonal matrix, such that:

(2) $$\begin{array}{l} d_{ij}=\{\begin{array}{cc} \frac{1}{\sum_{j}h_{ij}} & \text{If }i=j\\ \text{0} & \text{Otherwise} \end{array} \end{array}$$

After the convergence, *D*_*i*+1_ was considered as the propagated mutation profile that has values between zero and one.

### 2.2. Clustering Method

To cluster propagated mutation profiles, we used DEC method (Xie et al., 2016). Suppose we have *n* tumors with the feature vectors *x*_*i*_ in space *X* with *m* dimension that should be grouped to *k* clusters with centers μ_*j*_, *j* = 1, …, *k*. Instead of clustering the data in the initial space *X*, the data are mapped to the latent feature space *Z* by a nonlinear function *f*_θ_:*X* → *Z*, where θ is a set of trainable parameters. Usually, in order to avoid the curse of dimensionality, the dimension of *Z* is less than *m*. A deep neural network can be used to implement *f*_θ_, because of its theoretical function approximation characteristics (Hornik, 1991), and the capabilities in learning features (Bengio et al., 2013).

DEC is an iterative method, which learns cluster assignments and feature embedding simultaneously. In each iteration, the cluster centers ${\left\{\mu_{j}\in Z\right\}}_{j=1}^{k}$ as well as parameters θ are updated. This algorithm consists of two parts:

1. Parameter initialization using a stacked auto-encoder (SAE) (for θ) (Suk et al., 2015) and k-means algorithm (for centroids).
2. Parameter optimization that contains the alternative iteration of two steps: calculation of the auxiliary target distribution function, and updating the parameters using minimization of the Kullback–Leibler divergence (KLD).

In the initialization phase, the SAE is used to learn the feature embedding in an unsupervised manner. The SAE in this paper consists of two auto-encoders. Every auto-encoder has two layers as follows:

(3) $$\begin{array}{l} u=f\left(w_{1}\left(Dropout\left(x\right)\right)+b_{1}\right)y=g\left(w_{2}\left(Dropout\left(u\right)\right)+b_{2}\right) \end{array}$$

where Dropout function (Baldi and Sadowski, 2013) randomly sets some of input elements to zero, *f* is the encoder function, *g* is the decoder function, *w*_*i*_ is the weight of *i*th layer, and *b*_*i*_ is the bias of *i*th layer. The parameter set θ = {*w*_1_, *w*_2_, *b*_1_, *b*_2_} is learned in order to minimize the loss function $\|y-x{\|}_{2}^{2}$. After learning the first auto-encoder, the output of encoder (*u*) is regarded as the input of the second auto-encoder. When the SAE was trained, the feature vector *x*_*i*_ could be embedded to the latent feature *z*_*i*_ by applying the first and second encoders on it.

Next, a clustering layer is added after the encoder layers to cluster the latent features. The cluster centers (μ_*j*_) are initialized by running k-means on the latent features. The weights of the clustering layer were initialized by cluster centers.

In the optimization part, the latent features and clustering assignments are improved using alternating two following steps. In the first step, the latent feature (*z*_*i*_) is softly assigned to cluster center (μ_*j*_) with probability *q*_*ij*_:

(4) $$\begin{array}{l} q_{ij}=\frac{{\left(1+\|z_{i}-\mu_{j}{\|}^{2}\right)}^{-1}}{\sum_{j^{\prime }}{\left(1+\|z_{i}-\mu_{j^{\prime }}{\|}^{2}\right)}^{-1}} \end{array}$$

In the second step, the KLD between soft assignment distribution (*q*_*ij*_) and an auxiliary distribution (*p*_*ij*_) is calculated.

(5) $$\begin{array}{l} KLD\left(P\|Q\right)=\underset{i}{\sum }\underset{j}{\sum }p_{ij}\text{log}\frac{p_{ij}}{q_{ij}} \end{array}$$

The auxiliary distribution is defined as:

(6) $$\begin{array}{l} p_{ij}=\frac{q_{ij}^{2}/f_{j}}{\sum_{j^{\prime }}q_{ij^{\prime }}^{2}/f_{j^{\prime }}} \end{array}$$

where $f_{j}=\underset{i}{\sum }q_{ij}$ are the soft cluster frequencies. Then, the cluster center (μ_*j*_) and latent feature (*z*_*i*_) are updated in order to minimize the KLD using the stochastic gradient descent (Bottou, 2012).

These two steps are iterated until the convergence. The convergence criterion is satisfied when the assigned clusters to samples in two subsequent iterations are changed in <0.001 portion of data.

**Figure 1:**
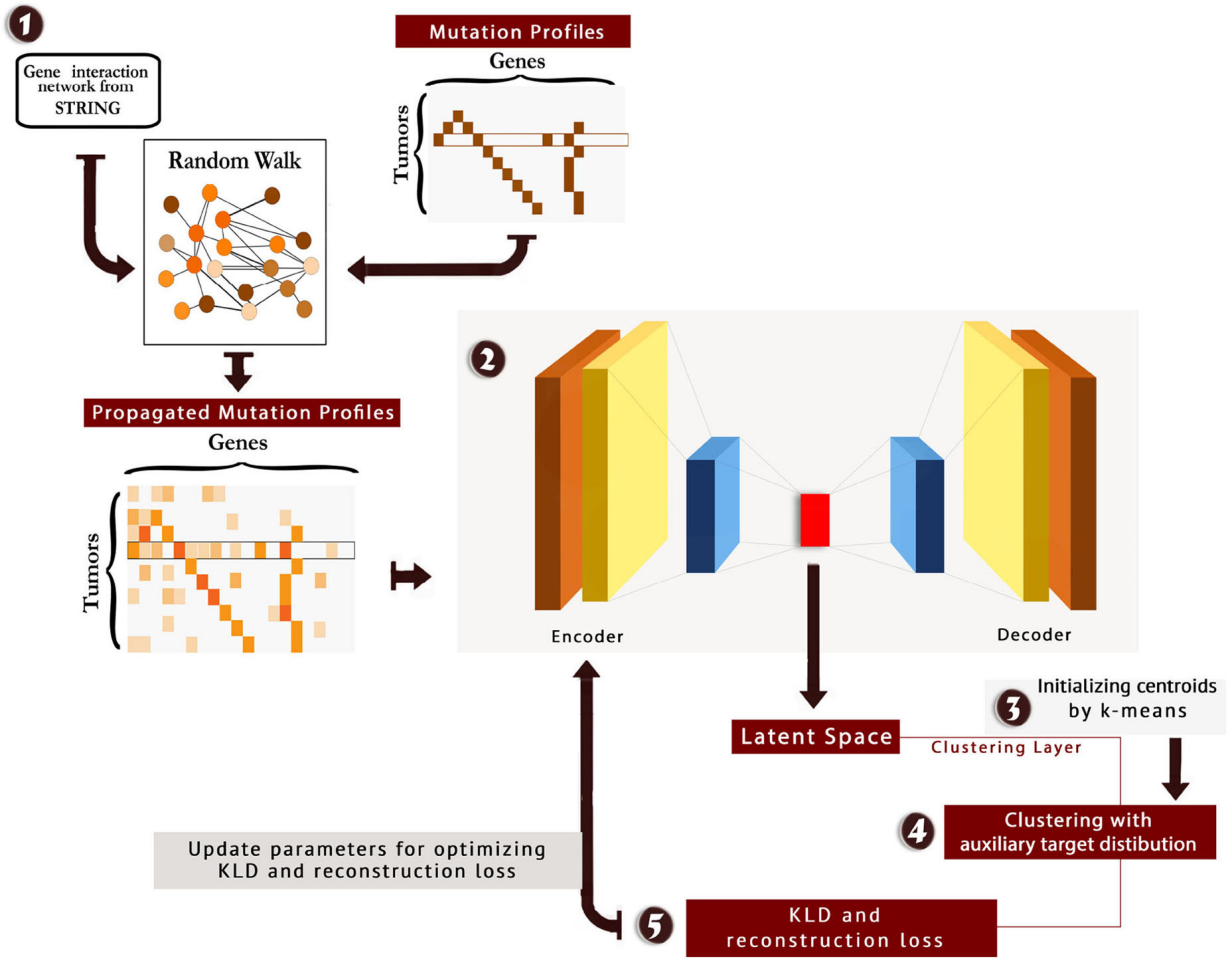
The scheme of MSDEC (discovering Molecular Subtypes by using Deep Embedded Clustering). **1**: The gene interaction network is obtained from STRING, and the nodes in the network are labeled based on the mutation profile of tumors. Applying random walk on this network yields propagated mutation profiles. **2**: The propagated mutation profiles are mapped to a latent space. **3**: A clustering layer is appended after encoder layers. The cluster centroids are initialized using k-means. **4**: The tumors are clustered using the auxiliary target distribution. **5**: The Kullback–Leibler divergence (KLD) and reconstruction loss are calculated, and the parameters are updated to minimize KLD and reconstruction loss.

We tuned hyperparameters of the model, and the best number of neurons in the stacked auto-encoder layers was 514, 500, 200, 500, and 514, respectively. Moreover, the best number of neurons for clustering layer was found to be 4. The scheme of the method is presented in Figure 1. Also, the code and material of the method are available at: https://github.com/nrohani/MolecularSubtypes.

### 2.3. Finding the Best Number of Clusters

The clustering method requires the number of clusters (*k*) as the input. For selecting the best number of clusters, the clustering algorithm was implemented with different values of *k*. There are some appropriate criteria to compare results and choose the best number of clusters.

**Figure 2:**
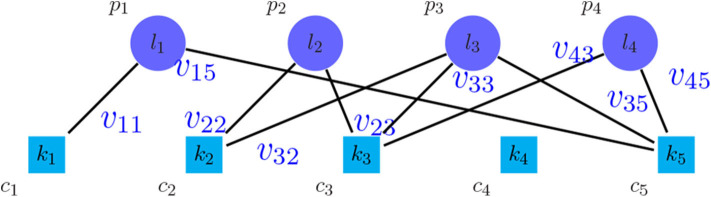
Bipartite graph between the method to be evaluated and PAM50.

An approach to find the number of clusters is to evaluate the clustering based on microarray-based classes (PAM50) (Parker et al., 2009) as the prior information. For this purpose, a weighted bipartite graph *G* was formed, where the nodes of one part were the clusters of PAM50, represented by *p*_*i*_ symbols, and the nodes of another part were the discovered clusters, represented by *c*_*j*_ symbols. We weighted the edge (*p*_*i*_, *c*_*j*_), represented by *v*_*ij*_, which shows the number of tumors shared between the clusters *p*_*i*_ and *c*_*j*_. Moreover, the vertices *p*_*i*_ and *c*_*j*_ were labeled by the their sizes, represented by *l*_*i*_ and *k*_*j*_, respectively. Figure 2 shows the general scheme of such graph. After creating the graph, the following metrics were calculated in order to find the best number of clusters:

(7) $$\begin{array}{l} PPV=\frac{\sum_{j=1}^{K}{\text{max}}_{i}\;v_{ij}}{\sum_{i=1}^{L}\sum_{j=1}^{K}v_{ij}} \end{array}$$

(8) $$\begin{array}{l} SN=\frac{\sum_{i=1}^{L}{\text{max}}_{j}\;v_{ij}}{\sum_{i=1}^{L}l_{i}} \end{array}$$

(9) $$\begin{array}{l} ACC=\sqrt{SN\times PPV} \end{array}$$

Brohee and Van Helden (2006) have introduced these criteria. *ACC* is the geometric mean of PPV and SN; thus, it is more comprehensive than PPV and SN.

Another important criterion is the MMR (Brohee and Van Helden, 2006). For calculating this criterion, graph *G* was made, and the weights on the edges (*v*_*ij*_) were calculated based on the threshold θ and the affinity score *NA*(*p*_*i*_, *c*_*j*_) as follows:

(10) $$\begin{array}{l} v_{ij}=\{\begin{array}{cc} NA\left(p_{i},c_{j}\right) & NA\left(p_{i},c_{j}\right)\geq \theta \\ 0 & \left(p_{i},c_{j}\right)<\theta \end{array} \end{array}$$

(11) $$\begin{array}{l} NA\left(p_{i},c_{j}\right)=\frac{|p_{i}\cap c_{j}{|}^{2}}{\left|p_{i}\|c_{j}\right|} \end{array}$$

MMR was defined as follows:

(12) $$\begin{array}{l} MMR=\frac{\sum_{v_{ij}\in Match_{w}\left(P,C,\theta \right)}v_{ij}}{\left|P\right|} \end{array}$$

where $Match_{w}\left(P,C,\theta \right)$ is the maximum weighted matching of *G*.

The discussed criteria compare the methods qualitatively. Another approach for comparison is the quantitative evaluation. We constructed a graph similar to the graph made for computing MMR. Then, we ignored the weight of the edges. Let Match(P,C,θ) to be the maximum non-weighted matching of this graph. Maddi et al. (2019) have introduced the following set of criteria:

(13) $$\begin{array}{l} N_{p}^{+}=|\left\{p_{i}|\;\exists c_{j},\;NA\left(p_{i},c_{j}\right)\geq \theta ,\;\left(p_{i},c_{j}\right)\in Match\left(P,C,\theta \right)\right\}| \end{array}$$

(14) $$\begin{array}{l} N_{c}^{+}=|\left\{c_{j}|\;\exists p_{i},\;NA\left(p_{i},c_{j}\right)\geq \theta ,\;\left(p_{i},c_{j}\right)\in Match\left(P,C,\theta \right)\right\}| \end{array}$$

(15) $$\begin{array}{l} Precision^{+}=\frac{N_{p}^{+}}{\left|P\right|} \end{array}$$

(16) $$\begin{array}{l} Recall^{+}=\frac{N_{c}^{+}}{\left|C\right|} \end{array}$$

(17) $$\begin{array}{l} F-measure^{+}=\frac{2\times Precision^{+}\times Recall^{+}}{Precision^{+}+Recall^{+}} \end{array}$$

*F* − *measure*^+^ is the harmonic mean of *Precision*^+^ and *Recall*^+^; thus, *F* − *measure*^+^ is more meaningful than *Precision*^+^ and *Recall*^+^. All the mentioned criteria are in the [0, 1] range.

One of the most comprehensive criteria in this issue is the AUMF (Maddi et al., 2019), which combines qualitative and quantitative attitudes. In fact, in this criterion the area under the curve (*MMR* + *Fmeasure*^+^, θ) is considered as a clustering measure called AUMF, which is in the [0, 2] range.

We executed DEC with the different numbers of clusters, and the results show that the best number of clusters is four (see Supplementary Figures 1, 2). Also, to evaluate the performance of the DEC method, this method was compared with other popular and common clustering methods such as hierarchical clustering (*HC*), k-means clustering, and spectral clustering (*SPC*) (Von Luxburg, 2007). DEC achieved better performance in comparison with other clustering methods.

### 2.4. Supervised Classification for New Tumors

Using the discovered breast cancer subtypes, we labeled each tumor with its discovered subtype and proposed a supervised classifier to understand how accurate the subtypes of new breast tumors can be predicted based on their somatic mutations. With this classifier, one can predict the subtype of a new patient using the somatic mutation profile as input. Five common machine learning classifiers were executed, namely, RF, support vector machine (SVM), multi-layer perceptron (MLP), naïve bayes (NB), and k-nearest neighbors (KNN) to classify the tumors into *k* subtypes ${\left\{C_{i}\right\}}_{i=1}^{k}$.

Due to the best results of RF (see section 3.6) in the supervised classification of tumors as well as its efficient application in feature selection, the RF was used to find important genes for classification. After training the RF, the importance of features can be calculated by considering the effect of using the features in reducing loss function (in this study, we used the Gini index as the loss function). In other words, the feature importance is the average reduction in loss function that induced by that feature. Then, the features with the importance of more than 0.01 were selected. The selected genes have the highest importance in detecting breast cancer subtypes.

## 3. Results

After clustering tumors using MSDEC method, four clusters were obtained with the following sizes:

- Subtype 1 (*Primary* subtype): 182 tumors,
- Subtype 2 (*Progressive* subtype): 82 tumors,
- Subtype 3 (*Proliferous* subtype): 499 tumors,
- Subtype 4 (*Perilous* subtype): 98 tumors.

**Figure 3:**
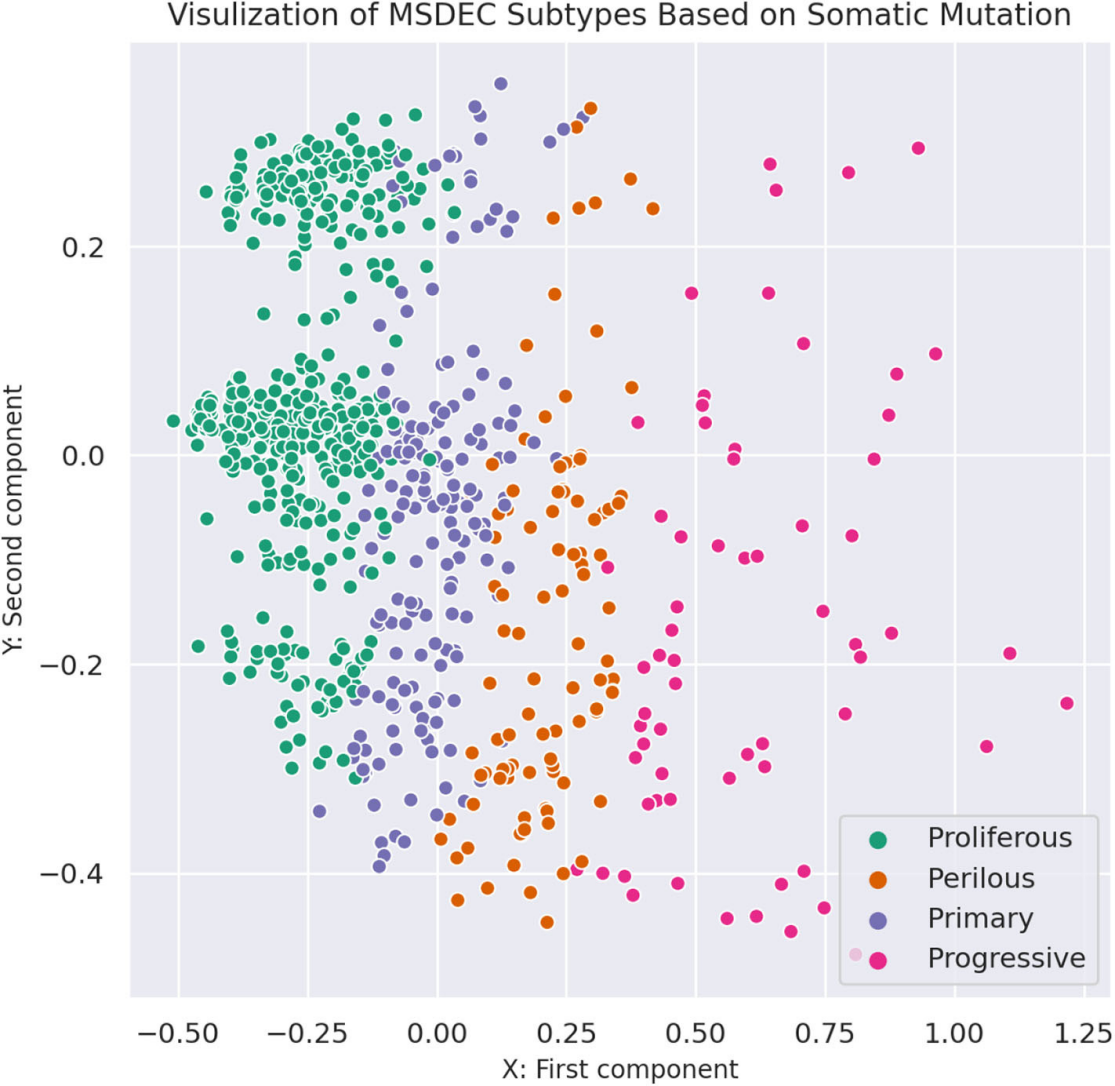
Visualization of MSDEC subtypes based on the somatic mutation profile of tumors. The axes are the first two principal components of propagated mutation profiles.

Figure 3 shows the illustration of the MSDEC subtypes. To visualize the tumors based on their mutation profile in a 2D space, we used principal component analysis (PCA) and obtained the first two principal components. Therefore, each tumor with a vector of length *n* representing the mutation status of the genes can be mapped to a 2D space using the first and second principal components. In Figure 3, the tumors are colored based on their assigned subtypes using MSDEC. It can be seen that the subtypes assigned by MSDEC are highly separable in this space. Precisely, all the tumors belonging to *Proliferous* subtype (green circles) are located at left, then *Primary* tumors (purple circles) are located at the right of them. The *Perilous* tumors are placed at the left side of *Primary* tumors. Moreover, *Progressive* tumors are settled at the right of the figure. The location of each subtype is specified and can be separated easily from the other subtypes. This figure shows that MSDEC subtypes have high separability.

To further investigate the discovered subtypes, we conducted the following evaluations.

### 3.1. Finding the Gene Signature for Each Subtype

One of the efficient evaluations is finding influential genes in each subtype. This evaluation is essential in two ways. First, it is possible to examine the biological significance of the clustering method; second, these genes can be considered as the candidates for the therapeutic purposes in each subtype's patients. For this purpose, the Fisher's exact test was used to find each subtype's gene signature. In the gene signature list, the top 50 genes with the *p*-value lower than 0.05 were considered and shown in Supplementary Figures 3–6. By investigating the top genes, one can conclude that the subtypes' key genes are different; thus, these genes can be suitable clues for choosing the treatment for the patients in each subtype. The gene interaction subnetwork of each subtype is obtained by enriching the subtype's gene signature into STRING database. The subnetwork of each subtype is illustrated in Supplementary Figure 7.

Many vital genes were found in the gene signature of the *Primary* subtype. One of them is *CDH*1, which produces E-cadherin protein. This protein is responsible for cell adhesion. Lacking E-cadherin allows the cancer cells to detach quickly and spread over the body and metastasize^1^. *CBFB* is another significant gene for *Primary* subtype. It encodes a transcription factor, which makes a complex by attaching to *RUNX*1^2^. This complex can transcriptionally repress the oncogenic *NOTCH* signaling pathway (Malik et al., 2019). *TBX*3 is a substantial gene in *Primary* subtype, which is needed for normal breast development^3^. Previous studies have shown that *TBX*3 leads to cell proliferation and suppresses apoptosis. *TBX*3 is regarded as a biomarker for breast cancer and has high importance in breast cancer diagnosis and treatment (Yarosh et al., 2008; Krstic et al., 2016). Another important gene in *Primary* subtype is *CTCF*, which encodes a transcription factor called zinc-finger. Studies have indicated that the mutation in *CTCF* is associated with the onset of breast cancer, prostate cancer, and Wilms' tumors (Oh et al., 2017), suggesting that this subtype mainly contains the tumors in early stages.

Many important genes such as *ERBB*2, *TP*53, *BRAF*, and *GNAS* are presented in the gene signature of the *Progressive* subtype. One of the driver genes in breast cancer is *ERBB*2, which is an indicator of tumor invasion (Revillion et al., 1998). Mutations and overexpression of this oncogene show the tendency of a tumor mass to become invasive, which may lead to the poor prognosis. The *BRAF* gene encodes a protein that helps transmit chemical signals from outside the cell to the cell's nucleus. This protein is responsible for regulating cell growth, proliferation, differentiation, migration, and apoptosis. Somatic mutations in this oncogene are prevalent in numerous cancers such as breast cancer, leading to the growth of cancerous cells^4^. The *TP*53 gene also is mutated in about 20 − −40% of breast cancer patients. It is useful to note that the mutation frequency is higher in patients with recurrent breast cancer (Norberg et al., 2001). Another essential gene for *Progressive* subtype is *GNAS*. The *GNAS* gene encodes the stimulatory alpha subunit of the *G* protein complex, which triggers a complicated network of signaling pathways that affect multiple cell functions by regulating the activity of hormones. This gene is known to be mutated in 0.74% of all cancers such as breast invasive ductal carcinoma, colon adenocarcinoma, lung adenocarcinoma, and rectal adenocarcinoma, in which invasive breast carcinoma has the highest frequency of mutations^5^. Therefore, the *Progressive* subtype is more invasive because its significant genes are mostly mutated in invasive cancers. The probability of the poor prognosis and metastasis may be high in this subtype.

The *Proliferous* subtype contains many important genes, such as *NOTCH*, *KRAS*, *PTEN*, and *WHSC*1*L*1. The *NOTCH* family genes, including *NOTCH*1, *NOTCH*2, *NOTCH*3, and *NOTCH*4, are highly expressed in breast cancer patients. These genes play an important role in the differentiation, proliferation, and cell cycle (Wang et al., 2011). About 80% of cancers have estrogen receptors, which are treated with anti-estrogen drugs. One of the leading causes of death in such patients is their resistance to anti-estrogen drugs. Estrogen pathways have a positive association with anti-estrogen drug resistance in ER-positive breast cancers by suppressing *NOTCH*1 (Hao et al., 2010). The *KRAS* gene produces the *K* − *Ras* protein, which affects cell proliferation, differentiation, and apoptosis^6^. The mutations of *KRAS* cause the production of abnormal *K* − *Ras* protein that leads to uncontrolled cell proliferation. Somatic mutations in this oncogene are substantial in different cancers, including breast cancer, papillary thyroid carcinoma (PTC), oral squamous cell carcinoma (OSCC), and gastric cancer (Sanaei et al., 2017). *WHSC*1*L*1 provides instructions for making *histone* − *lysineN* − *methyltransferase* NSD3 enzyme. It may involve in carcinogenesis, which is amplified in several cancers such as lung cancer and head and neck cancer^7^. Previous studies have suggested a close relation between *WHSC*1*L*1 mutation and breast cancer initiation and progression. The mutated *WHSC*1*L*1 is regarded as a candidate target for the treatment of breast cancer (Liu et al., 2015). *PTEN* gene encodes a tumor suppressor, which suppresses rapid and uncontrolled cell division. It also controls cell migration and adhesion. Somatic mutations of *PTEN* lead to the uncontrolled growth and division of cancerous cells. These mutations are involved in breast cancer (Zhang et al., 2013). Previous studies have shown that mutation in *PTEN* is a factor of resistance to trastuzumab (Herceptin) drug, which is used for the treatment of breast cancer^8^.

Many essential genes are found among the gene signature of *Perlious* subtype such as *MYC*, *ITSN*1, *KDM*5*C*, and *TEP*1. One of the critical regulators of cell growth, proliferation, metabolism, differentiation, and apoptosis is *MYC*. Mutations of this gene have many roles in the development and progression of breast cancer, activation of oncogenes, and inactivation of tumor suppressors (Xu et al., 2010). *TEP*1 is one of the telomeres length genes that is linked with cancer (Pellatt et al., 2013). Previous studies have provided evidence for the relation of mutations in *TEP*1 and breast cancer (Savage et al., 2007). *ITSN*1 provides instructions for making a cytoplasmic membrane-associated protein. It is associated with the actin cytoskeleton reconstruction in breast cancer (Xie et al., 2019). *KDM*5*C* controls the transcription and chromatin remodeling regulation. TCGA has identified *KDM*5*C* mutation as a cancer driver mutation in the genes encoding the histone demethylases. Studies on oncometabolite have shown that the *KDM*5*C* is involved in cancer-related metabolic reprogramming and the tumor suppression (Chang et al., 2019). Thus, mutations of this oncogene are associated with tumor progression. It is mutated in 0.22% of all cancers, such as breast invasive ductal carcinoma, lung adenocarcinoma, prostate adenocarcinoma, and high-grade ovarian serous adenocarcinoma. Among these cancers, mutations of *KDM*5*C* are the most prevalent in invasive breast carcinoma^9^.

### 3.2. Survival Analysis

**Figure 4:**
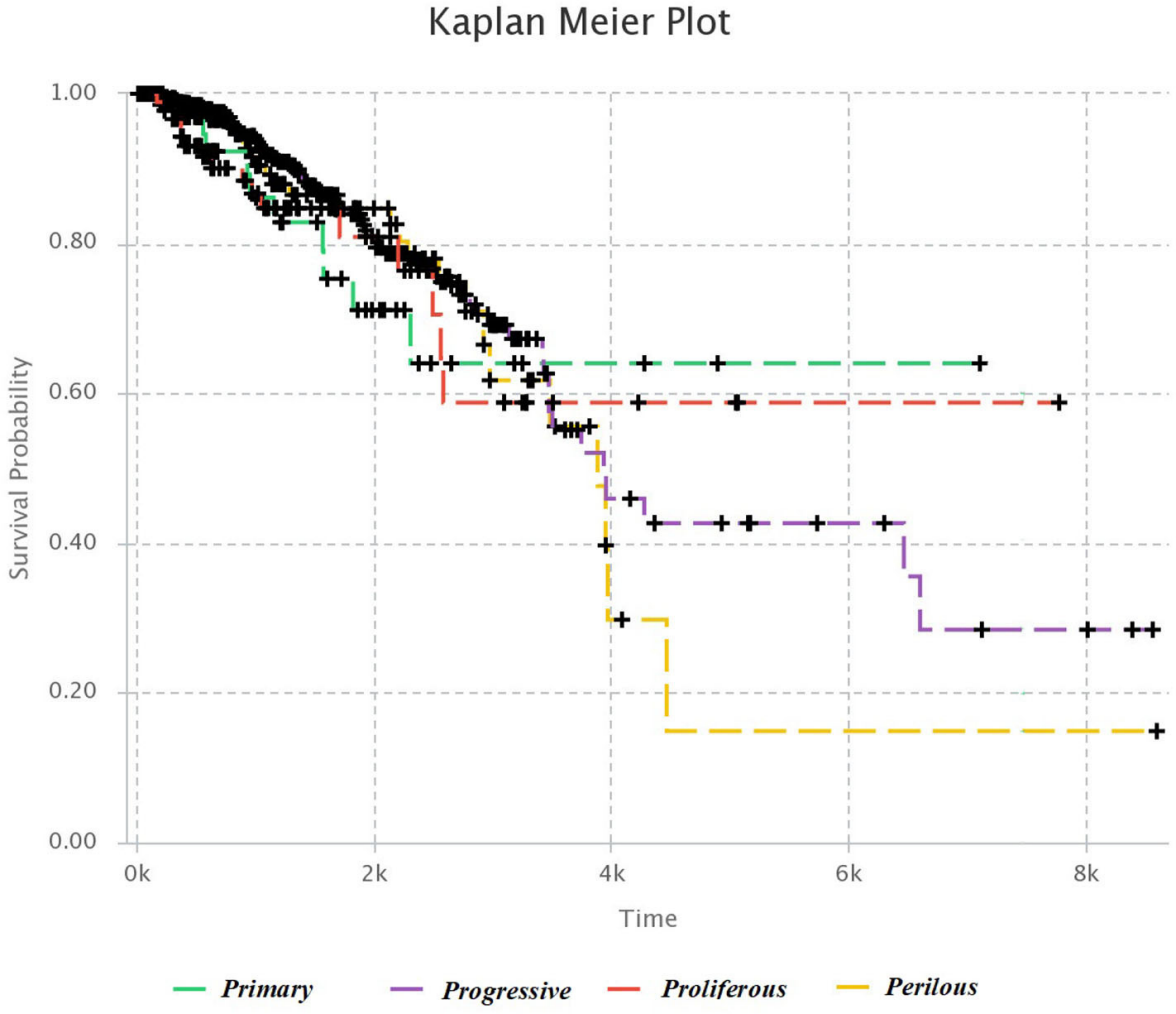
Kaplan–Meier survival diagram. Each line shows the percentage of survived patients with a subtype of breast cancer after a specific time.

We used Kaplan–Meier estimator (Kleinbaum and Klein, 2012) for survival analysis in each subtype, which is shown in Figure 4. The horizontal axis is the time after diagnosis, and the vertical axis represents the percentage of patients. The percentage of patients that are survived after specific days are plotted, and colored lines link the patients with the same subtype. The lower plot of survival demonstrates the more hazardous subgroup of people.

It was mentioned in section 3.1, that *Progressive* subtype is invasive, due to the set of significant genes in this subtype. This issue is consistent with survival analysis. It can be seen that the *Progressive* subtype has the lowest survival.

Moreover, the cox hazard regression was computed for further survival analysis. The diagram of cox hazard regression is presented in Supplementary Figure 8. To examine the significance of subtypes in predicting the patient's survival, chi-squared test was used, which shows that subtype is an essential feature in cox hazard regression (*p* = 0.00475). This analysis indicates that MSDEC subtypes have a significant correlation with the hazard rate.

### 3.3. Protein Complexes Analysis

**Figure 5:**
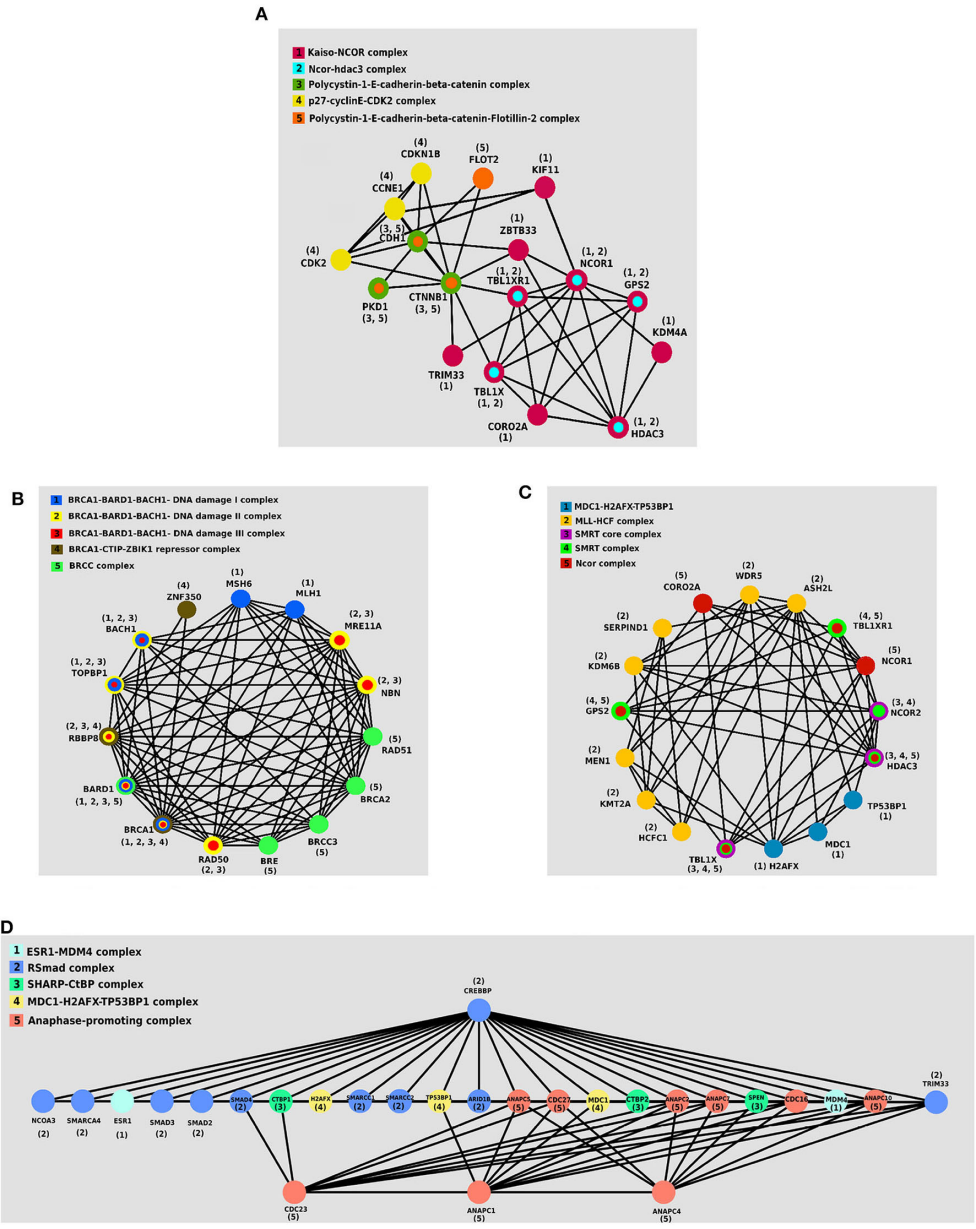
The protein–protein interaction (PPI) networks of protein complexes in discovered subtypes. The proteins assigned to the same complex are shown with the same color and labeled with the same number. **(A)** Five protein complexes in *Primary* subtype. **(B)** Five protein complexes in *Progressive* subtype. **(C)** Five protein complexes in *Proliferous* subtype. **(D)** Five protein complexes in *Perilous* subtype.

We investigated the essential protein complexes in each subtype because most of the cell activities are carried out by protein complexes. The gene signature of each subtype was entered to the *iRefWeb* (Turner et al., 2010) website; then, the sorted complexes of each subtype were obtained (see Supplementary Tables 1–4). More information on these complexes is available in the *CORUM* database (Ruepp et al., 2009). Figures 5A–D visualizes five protein complexes in the *Primary*, *Progressive*, *Proliferous*, and *Perilous* subtypes, respectively. The nodes of these graphs represent the proteins that are involved in five complexes, which are obtained from *CORUM* database (Ruepp et al., 2009). The interactions between proteins were obtained from *STRING* database (Szklarczyk et al., 2016) and were shown by the edges in these graphs. The numbers beside the nodes represent the complexes that the protein are cooperating in them. Moreover, the nodes are colored based on their complexes.

One of the notable complexes in the *Primary* subtype is the *p*27 − *cyclinE* − *CDK*2 complex, which contains two *CDK*2 and *CDKN*1*B* genes. This complex is involved in cell cycle regulation, cell cycle control, and *DNA* processing. One of the crucial regulators of the cell cycle is *CDKN*1*B*, which inhibits *G*1/*S* by clinging to *CDK*2 and suppressing it. Overexpression of *CDKN*1*B* gene in specific cancer cells prevents DNA replication and tumorigenesis, whereas its deficiency plays an inhibitory role in human cancers and decreases the chance for developing breast, prostate, colon, lung, and esophagus cancers (Xu et al., 2007).

*BRCC* complex includes the genes *BRCA*1, *BRCA*2, *BRCC*3, *RAD*51, and *BRE*, which is among the influential complexes in the *Progressive* subtype. The function of the *BRCA*1 gene in *DNA* repair and cell cycle control in response to *DNA* damage is regulated by other complexes. Interaction of *BRCA*1 with *RAD*51 has a direct impact on the double-strand breaks of *DNA* (Christou and Kyriacou, 2013). Not only has *ERCC* complex a direct interaction with *TP*53 in the destruction of *DNA*, but also it causes the displacement of *DNA*. Recently, the expressions of two new members of the complex, namely *BRCC*36 and *BRCC*45, have been discovered in breast cancer cells (Dong et al., 2003).

The set of *TBL*1*X*, *HDAC*3, and *NCOR*2 genes together make the *SMRT* complex, which plays a vital role in *Proliferous* tumors. The *SMRT* complex is both an activator and a suppressor of the estrogen receptor-α (*ER* − α), which its overexpression in breast cancer can make therapeutic outcomes more complicated. The activity of this complex inhibits the regulated cell death using the genes involved in apoptosis. This complex activates the anti-apoptotic genes and suppresses the pro-apoptotic genes. Thus, by activating multiple pathways, this complex leads to the progression and proliferation of breast cancer with declining apoptosis (Blackmore et al., 2014).

*ESR*1 − *MDM*4 complex that is consisted of two genes *ESR*1 and *MDM*4 proteins is essential in the *Perlious* subtype. The estrogen hormone receptor *ESR*1 is a nuclear hormone receptor that is expressed in approximately 70% of patients with breast cancer (Stanford et al., 1986). The expression of *MDM*4 gene is positively correlated with the expression of *ERα* in primary breast tumors. Also, *ERα* enhances the expression of *MDM*2 (Baunoch et al., 1996).

### 3.4. Clinical Examination

We investigated the relationship between each subtype and the clinical features such as *ER* status, *PR* status, *HER*2 status, TP53 status, and histopathological subtypes using the chi-squared test. The contingency tables of these analyses are shown in Supplementary Figures 9–13. The MSDEC subtypes have a significant correlation with the mentioned clinical features.

Supplementary Figure 9 shows the relation of the *ER* status with the MSDEC subtypes (*p* < 2.2*E* − 16 by chi-squared test and *p* = 1*E* − 06 by Fisher's exact test). By considering the results of two tests, it can be concluded that the *ER* status of tumors is not significantly independent of the MSDEC subtypes. Thus, MSDEC subtypes are related to this clinical factor. Moreover, it can be seen that the majority of tumors in *Primary* and *Proliferous* subtypes are mostly ER-positive.

The contingency table in Supplementary Figure 10, shows the relationship of the *PR* status with MSDEC subtypes. The *p*-values of the chi-squared test and Fisher's exact test on this table were 2.2*E* − 16 and 1*E* − 06, respectively. Therefore, the MSDEC subtypes are not significantly independent of the *PR* status of patients. The rate of *PR* positive is higher than *PR* negative in the *Primary* and *Proliferous* subtypes, while most tumors in the *Progressive* and *Perlious* subtypes are *PR* negative.

The contingency table in Supplementary Figure 11, was constructed to examine the association of *HER*2 status with the MSDEC subtypes. The *p*-values of the chi-squared test and Fisher's exact test in this table were 1.445*E* − 07 and 1*E* − 06, respectively, which indicate a significant relationship between the clinical status of *HER*2 and the MSDEC subtypes. It can also be carefully deduced from this table that the *Primary* and *Proliferous* subtypes are significant *HER*2 negative.

The contingency table that indicates the relation of the *TP*53 status with MSDEC subtypes is shown in Supplementary Figure 12. The *p*-values of the chi-squared test and Fisher's exact test on this table were 2.2*E* − 16. Therefore, the MSDEC subtypes are not significantly independent of the *TP*53 mutations in patients. One of the interesting points in this table is the low rate of *TP*53 mutations in *Proliferous* and *Primary* subtypes, which indicates a noninvasive and better diagnostic status for *Primary* and *Proliferous* tumors. Thus, the *Primary* and *Proliferous* subtypes include tumors that have a better prognosis. In the *Progressive* and *Perilous* subtypes, the mutations pattern of *TP*53 is reversed, and its mutated state is more prevalent than its wild type.

We examined the association of the MSDEC subtypes with the histopathological subtypes. The distribution of these two variables in relation to each other is shown in Supplementary Figure 13, which has *p* = 0.0001615 by the chi-squared test and *p* = 5.4*E* − 05 by the Fisher's exact test. As a result, there is strong evidence for the significant correlation between the two types of classification.

On the whole, the characteristics of the MSDEC subtypes can be summarized as follows.

*Primary* and *Proliferous* subtypes are consisted of tumors that are *ER*+ and *PR*+. The higher rate of *PR* positive than *PR* negative in the *Primary* and *Proliferous* subtypes indicate that most tumors in these two subtypes are *luminal* tumors. It can also be carefully deduced from the Supplementary Figure 11 that the *Primary* and *Proliferous* subtypes are significantly negative for *HER*2. These tumors have wild-type *TP*53, and one of their most significant genes is *CDH*1.

Moreover, *Progressive* and *Perilous* subtypes mostly contain tumors that are *PR*−. *TP*53, *ERBB*2, *BRCA*1, and *MYC* are the significant genes in *Progressive* and *Perilous* subtypes. Mutations of the *BRCA*1 and *MYC* genes exacerbate breast cancer (Xu et al., 2010). Additionally, high rate of *TP*53 mutations in these subtypes suggest that the *Progressive* and *Perilous* subtypes may have poor diagnostic status.

### 3.5. Comparison Between MSDEC and PAM50 Subtypes

We compared the MSDEC subtypes from somatic mutation with PAM50 subtypes obtained from micro-array data; thus, the following evaluations were conducted to investigate their similarities and differences.

**Figure 6:**
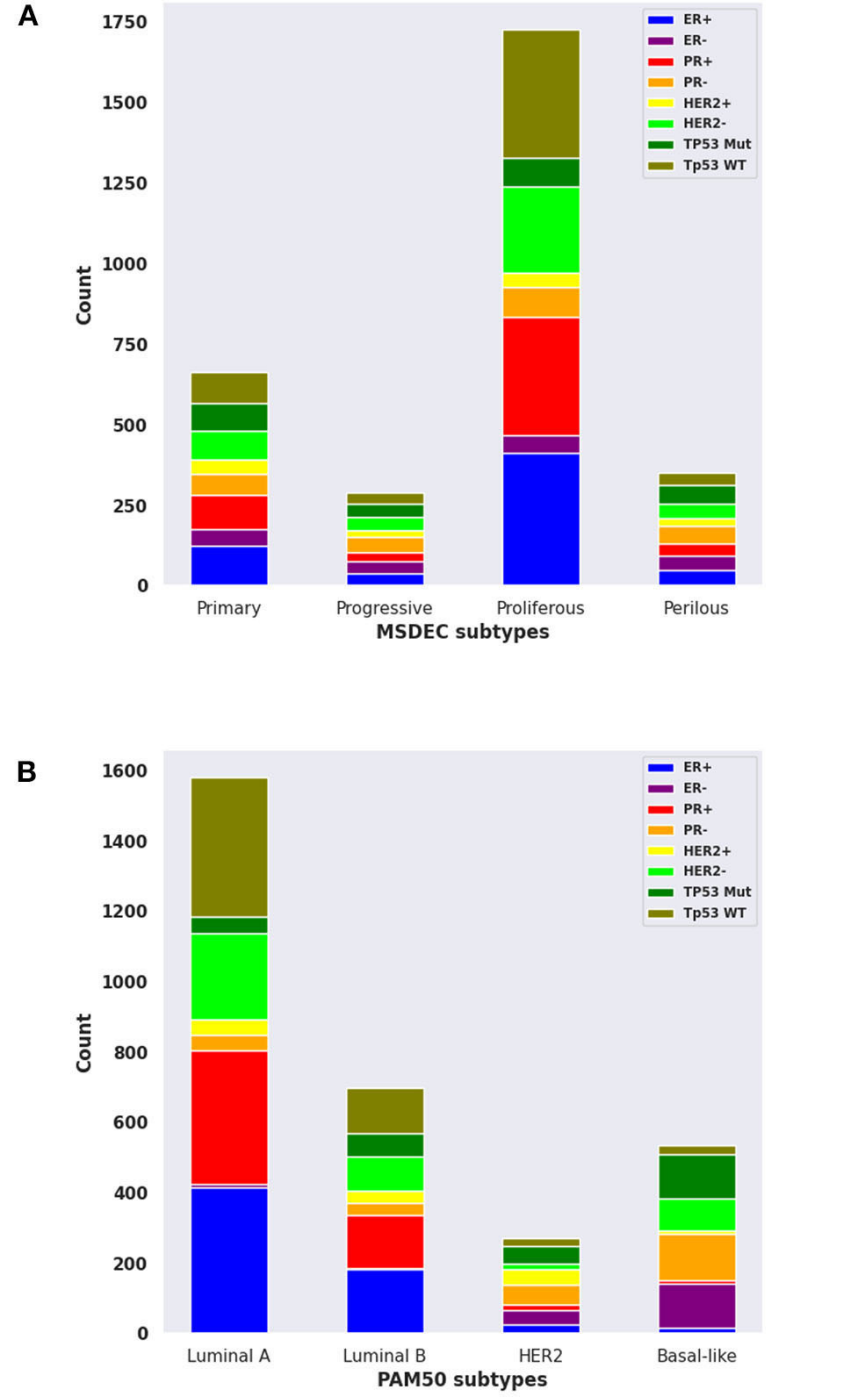
The composition of ER+/-, PR+/-, HER2+/-, and mutant or wild-type TP53 in MSDEC and PAM50 subtypes. **(A)** The number of tumors with ER+/-, PR+/-, HER2+/-, and mutant or wild-type TP53 in each MSDEC subtypes. **(B)** The number of tumors with ER+/-, PR+/-, HER2+/-, and mutant or wild-type TP53 in each PAM50 subtypes.

The contingency table in Supplementary Figure 14 shows the intersection of tumors between the MSDEC subtypes and PAM50 subtypes. It is noteworthy that this table is not static since the assignment of tumors to PAM50 subtypes changes dynamically (Pusztai et al., 2006; Gusterson, 2009; Weigelt et al., 2010; Vural et al., 2016). The dependency of these two clusterings was evaluated by using chi-squared test, which yielded *p* < 2.2*E* − 16, and Fisher's exact test, which led to *p* = 1*E* − 06. Moreover, the composition for each subtype with ER+/–, PR+/–, HER2+/–, and TP53 (mutated/wild type), and the PAM50 is visualized in Figures 6A,B, respectively.

Among the PAM50 subtypes, *luminal A* and *luminal B* are *HER*2 negative and *ER* positive. These tumors have a good prognosis and long survival. These subtypes are most similar to *Primary* and *Proliferous* subtypes due to the status of *ER*, *HER*2, and based on their prognosis and survival. Moreover, *Primary* and *Proliferous* tumors have wild-type *TP*53. One of their most significant genes is *CDH*1, which is highly expressed in the *luminal A* and *luminal B* subtypes, while it has low activity in *HER*2 − *positive* and *basal* − *like* subtypes (Zaha et al., 2019). However, the higher rate of *PR* positive than *PR* negative in the *Primary* and *Proliferous* subtypes may differ from *LuminalB* tumors.

Moreover, *basal* − *like* and *HER*2 subtypes mostly contains tumors that are *PR*−, which suggest that these two subtypes are more similar to *Progressive* and *Perilous* tumors. *TP*53, *ERBB*2, *BRCA*1, and *MYC* are the significant genes in *Progressive* and *Perilous* subtypes. Mutations of the *BRCA*1 and *MYC* genes exacerbate breast cancer (Xu et al., 2010). The *MYC* gene is highly expressed in the *basal* −*like* subtype of breast cancer, which is being targeted for treatment in these patients. Given the poor diagnostic status and high rate of *TP*53 mutations in the *basal* − *like* and *HER*2 subtypes, one can conclude that the *Progressive* and *Perilous* subtypes are related to the *basal* − *like* and *HER*2 subtypes (Xu et al., 2010).

To sum up, the *Primary* and *Proliferous* mostly contain *luminal A* and *luminal B* tumors, while the majority of tumors in *Progressive* and *Perilous* subtypes are *HER*2 − *positive* and *basal* − *like*. It is noteworthy that although the majority of tumors in *Primary* and *Proliferous* are *luminal A* and *luminal B*, numerous *HER*2 − *positive* and *basal* − *like* tumors are included in these two subtypes. A similar issue is true for *Progressive* and *Perilous* subtypes. Thus, the MSDEC subtypes are not fully matched with PAM50 subtypes. It is worth mentioning that PAM50 subtypes were obtained by clustering microarray data, whereas the MSDEC subtypes are the results of clustering the mutation profiles. Since applying different unsupervised methods on different features yield different results, it is obvious that the MSDEC and PAM50 subtypes are not the same.

**Figure 7:**
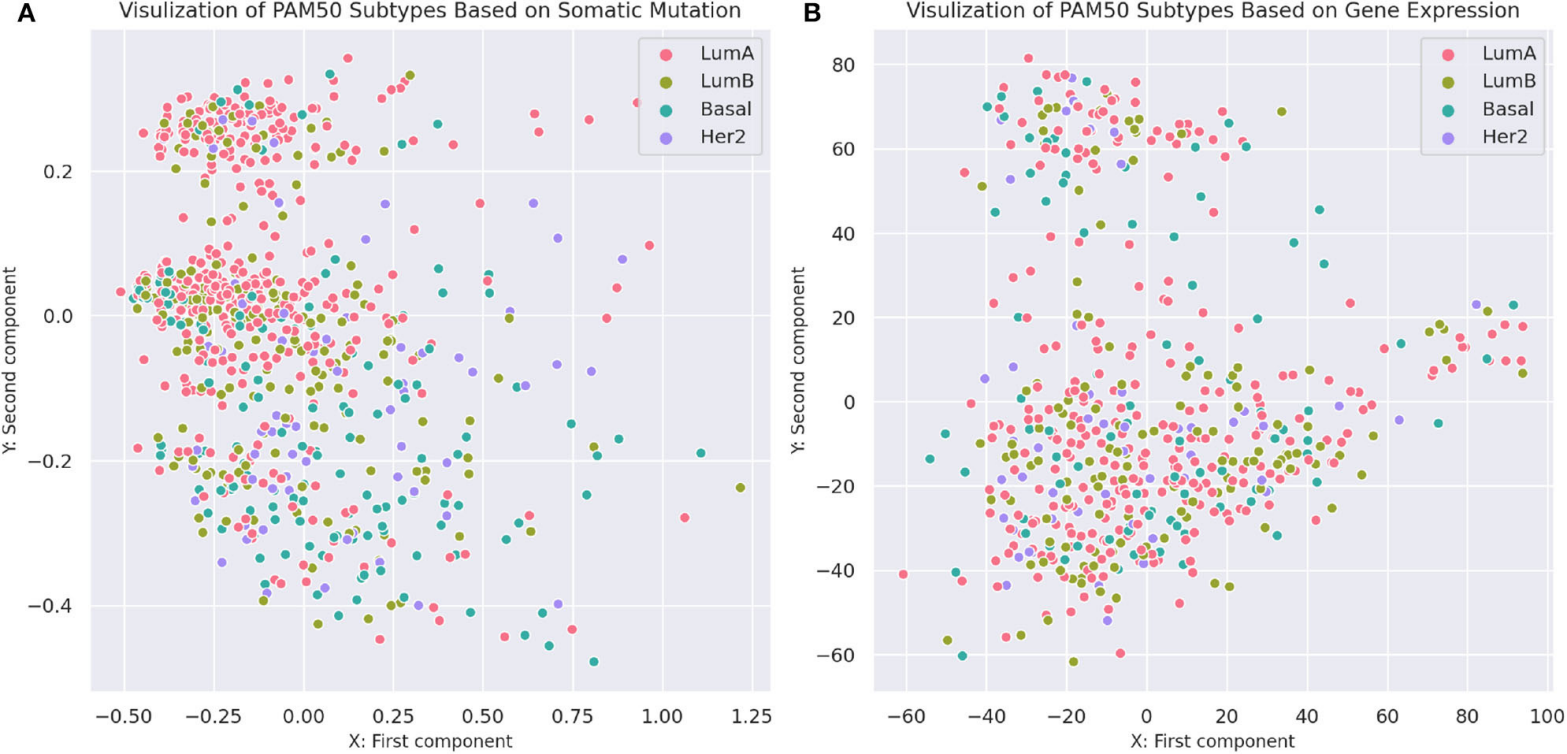
Visualization of PAM50 subtypes based on somatic mutation and gene expression profiles of the tumors. **(A)** Visualization based on somatic mutation profiles. The axes are the first two principal components of the propagated mutation profiles. **(B)** Visualization based on gene expression profiles. The axes are the first two principal components of gene expression profiles.

To compare the separability of subtypes identified by MSDEC and PAM50, we visualized the *PAM*50 subtypes in 2D space. To this aim, we used PCA to reduce the dimension of data and colored the tumors based on their subtypes. For the sake of simplicity in comparing subtypes identified by MSDEC and PAM50, we first applied PCA on the mutation profile of tumors, used the first two principal components to visualize the tumors, and colored them based on the PAM50 subtypes. Figure 7A shows the illustration of the PAM50 subtypes based on somatic mutation. One can figure out by the comparison of Figures 3A, 7 that the location of tumors are the same in these figures, while having different color scheme, one based on MSDEC and another based on PAM50 subtypes. In spite of Figure 3 that shows high separation in the MSDEC subtypes, the PAM50 subtypes in Figure 7A do not have favorable separation and all the subtypes seems to be mixed up in 2D space. Moreover, since PAM50 is clustering tumors based on gene expression, we plotted the tumors on the 2D space based on the first two principal components of the gene expression profiles to have a fair notion of the visualization of PAM50 subtypes. Figure 7B shows the illustration of *PAM*50 clusters based on gene expression. Same as in Figure 7A, the other illustrations of PAM50 subtypes in Figure 7B does not demonstrate high separability.

Moreover, we computed the silhouette criterion for assessing MSDEC and PAM50 clustering quantitatively. The silhouette criterion measures the difference between the similarity of a tumor to its own cluster (cohesion) compared to its similarity to other clusters (separation). The value of this criterion ranges from −1 to +1. The higher the silhouette, the better tumors are matched to their own clusters rather than other clusters. For a tumor *i* in cluster *C*_*k*_, the silhouette value is computed as formula 19.

(18) $$\begin{array}{l} s\left(i\right)=\frac{b\left(i\right)-a\left(i\right)}{\text{max}\left\{a\left(i\right),b\left(i\right)\right\}} \end{array}$$

where *a*(*i*) and *b*(*i*) are the cohesion and separation values for tumor *i*, which are calculated as follows:

(19) $$\begin{array}{l} a\left(i\right)=\frac{1}{|C_{k}|-1}\underset{j\neq i,j\in C_{k}}{\sum }d\left(i,j\right) \end{array}$$

(20) $$\begin{array}{l} b\left(i\right)=\underset{l\neq k}{\text{min}}\frac{1}{\left|C_{l}\right|}\underset{j\in C_{l}}{\sum }d\left(i,j\right) \end{array}$$

*d*(*i, j*) is the Euclidean distance between tumors *i* and *j*. The silhouette criterion for a clustering method is computed by averaging the *s*(*i*) values over all tumors. This criterion demonstrates that how tightly are the tumors in a cluster and how far are the tumors in diverse clusters. Therefore, this can be a measure for assessing the appropriateness of clustering methods. The computed silhouette criterion for MSDEC was 0.07011, while the computed silhouette criterion for PAM50 clusters based on gene expression and mutation profiles was 0.00956 and −0.00577, respectively. Comparison of the silhouette for MSDEC and PAM50 shows that MSDEC yields more appropriate subtypes.

### 3.6. Evaluation of Supervised Methods

Five classifiers, namely, RF, SVM, MLP, KNN, and NB, were compared using tenfold cross-validation. In tenfold cross-validation, the whole set of tumors was randomly divided into ten subsets with almost the same size. Then, one subset was put aside, and the model was trained with nine other subsets and evaluated with the remaining subsets. This process was repeated, such that each of the ten subsets was considered as the test data once. In this study, the tenfold cross-validation was repeated 100 times, and the average performance of the model was reported. The performance of the model was measured by standard evaluation criteria such as Accuracy, Sensitivity, Precision, F-measure, and AUC.

(21) $$\begin{array}{l} Accuracy=\frac{\sum_{i=1}^{k}\frac{TP_{i}+TN_{i}}{TP_{i}+TN_{i}+FP_{i}+FN_{i}}}{k} \end{array}$$

(22) $$\begin{array}{l} Precision=\frac{\sum_{i=1}^{k}TP_{i}}{\sum_{i=1}^{k}\left(TP_{i}+FP_{i}\right)} \end{array}$$

(23) $$\begin{array}{l} Recall=\frac{\sum_{i=1}^{k}TP_{i}}{\sum_{i=1}^{k}\left(TP_{i}+FN_{i}\right)} \end{array}$$

(24) $$\begin{array}{l} F-measure=\frac{2\cdot Precision\cdot Recall}{Precision+Recall} \end{array}$$

where *TP*_*i*_, *TN*_*i*_, *FP*_*i*_, and *FN*_*i*_ stand for the number of True Positives, True Negatives, False Positives, and False Negatives of class ${\left\{C_{i}\right\}}_{i=1}^{k}$. Since the values of Accuracy, Precision, Recall, and F-measure are dependent on the value of a threshold, we also evaluated methods using AUC, which is the area under the receiver operating characteristic (ROC) curve. The ROC curve plots True Positive Rate (TPR) vs. False Positive Rate (FPR). For each class *i*, *AUC*_*i*_ is the area under the curve plotting *TPR*_*i*_ vs. *FPR*_*i*_. Moreover, *AUC* for all classes is the area under the ROC curve of all classes, which is plotted with two approaches, namely, micro_average and macro_average. In micro_average, the ROC curve plots *TPR*_*micro*_ vs. *FPR*_*micro*_, while in macro_average, the ROC curve plots *TPR*_*macro*_ vs. *FPR*_*macro*_. AUC criterion indicates the efficiency of methods independent of the threshold value.

(25) $$\begin{array}{l} TPR_{i}=\frac{TP_{i}}{TP_{i}+FN_{i}} \end{array}$$

(26) $$\begin{array}{l} FPR_{i}=\frac{FP_{i}}{FP_{i}+TN_{i}} \end{array}$$

(27) $$\begin{array}{l} TPR_{macro}=\frac{\sum_{i=1}^{k}TPR_{i}}{k} \end{array}$$

(28) $$\begin{array}{l} FPR_{macro}=\frac{\sum_{i=1}^{k}FPR_{i}}{k} \end{array}$$

(29) $$\begin{array}{l} TPR_{micro}=\frac{\sum_{i=1}^{k}TP_{i}}{\sum_{i=1}^{k}TP_{i}+FN_{i}} \end{array}$$

(30) $$\begin{array}{l} FPR_{micro}=\frac{\sum_{i=1}^{k}FP_{i}}{\sum_{i=1}^{k}FP_{i}+TN_{i}} \end{array}$$

**Figure 8:**
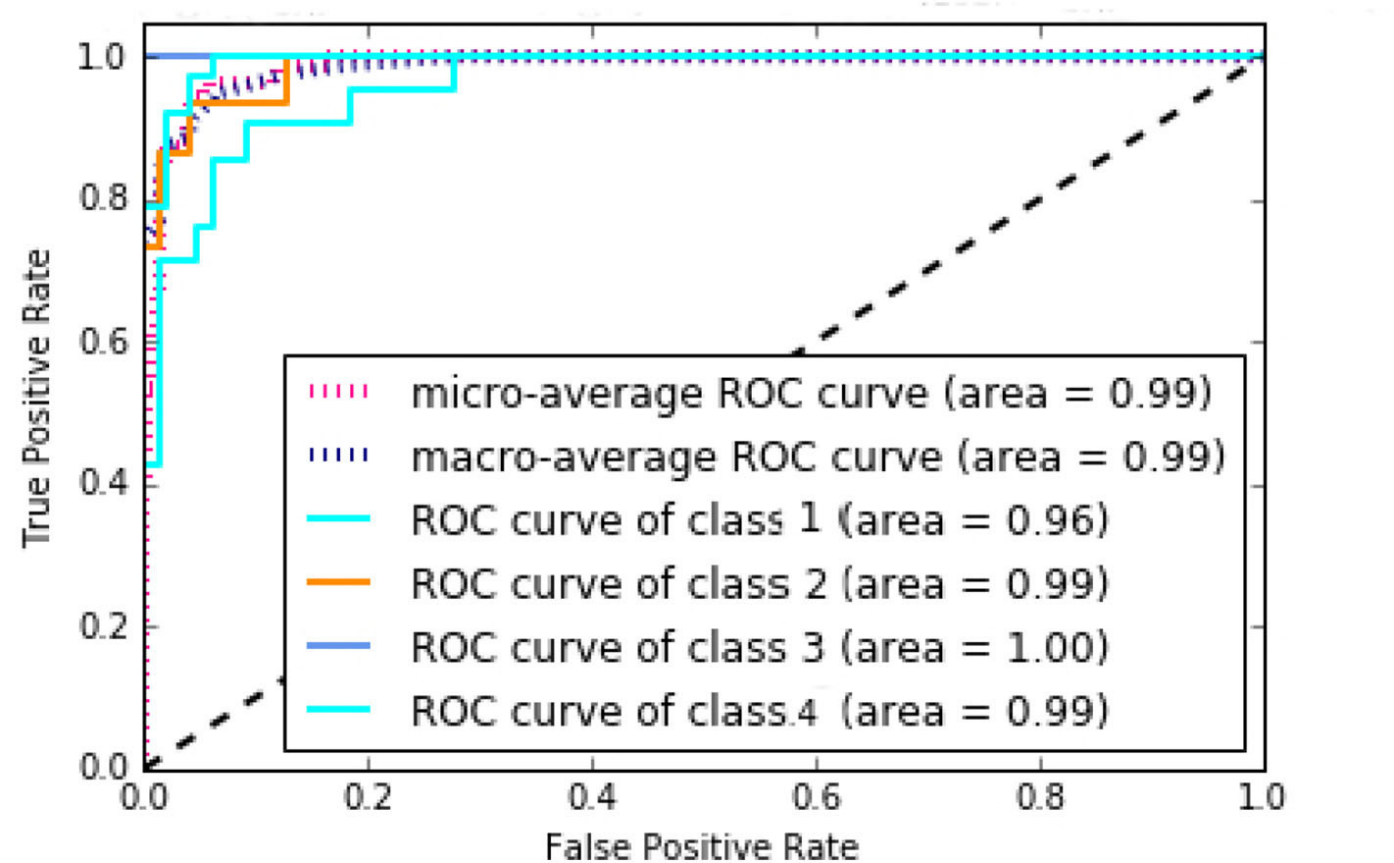
Area under the ROC curves of the random forest (RF).

According to Supplementary Figure 15, NB method has the worst performance, and SVM, KNN, and MLP have average performances. The best method with regard to all criteria is the RF with AUC of 99%, Accuracy of 86%, Precision of 90%, Recall of 85%, and F-measure of 87%, which has achieved great results. It can be concluded that the discovered subtypes by MSDEC method are separable; also, these subtypes can be predicted only by receiving mutations of 16 important genes for new tumors that were obtained using RF. The 16 important genes is as follows: *AKT*2, *CARD*11, *EIF*4*A*2, *FLNA*, *HNF*1*A*, *IDH*2, *LAMA*1, *LTBP*1, *MAP*2*K*1, *NCOR*2, *NOS*2, *PPP*1*R*12*A*, *PTPRU*, *SMC*1*A*, *TPR*, and *UPF*3*B*. The mutational frequency of 16 important genes in each subtype is shown in Supplementary Figure 16. Figure 8 shows the ROC curves of the RF classifier for each subtype. The value of *AUC* is excellent for each subtype and very close to one. However, the value of AUC for the *Proliferous* subtype is equal to one, which indicates that the model fits well on the tumors of the *Proliferous* subtype.

### 3.7. GSEA Enrichment

**Figure 9:**
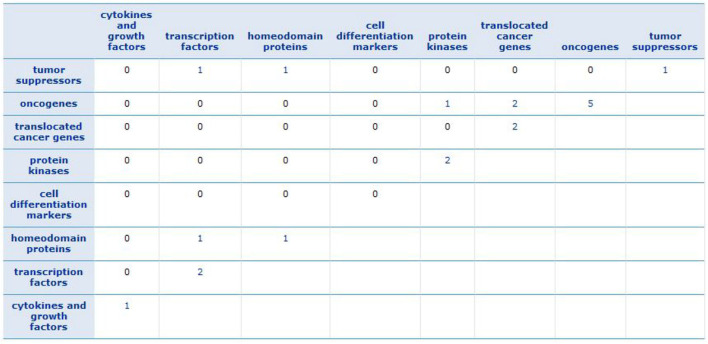
GSEA enrichment of 16 important genes. The numbers show how many of important genes are incorporated in each family.

To find a family of genes that are related to cancer, we enriched the gene signature of each subtype (see Supplementary Material) by Gene Set Enrichment Analysis (GSEA) tool (Subramanian et al., 2005). We recognized that the most of these genes belong to transcription factor and protein kinase gene families, which are known to be associated with the progression of breast cancer. The results are described in Supplementary Figures 17–20. Besides, Figure 9 shows the GSEA enrichment of 16 important genes, obtained using RF. It verifies that many of these genes are the most important genes in cancer.

## 4. Discussion

Cancer is a heterogeneous disease; so, accurate classification of cancer is crucial to find the appropriate treatment. Recent advances in molecular biology have provided high-quality and diverse data for the researchers. Recently, somatic mutation has attracted much attention in molecular cancer subtypes detection because it is more stable than other types of data and is commonly used for cancer treatment due to a large number of guidelines for single-gene mutations. In this study, the novel breast cancer molecular subtypes were presented using the profile of somatic mutations. Four discovered subtypes were obtained using network propagation with DEC. To analyze the characteristics of tumors in each subtype, we conducted numerous experiments, including finding gene signatures, protein complexes, gene families, and clinical features.

The results show that the *Primary* and *Proliferous* subtypes are mainly *ER*+, *PR*+, *HER*2−, and wild-type *TP*53; however, they have different important gene signature and protein complexes. Also, both of these subtypes contain the early stage and noninvasive tumors; the tumors in *Primary* have a higher probability of survival. Moreover, *Progressive* and *Perlious* subtypes are mainly *PR*− and have mutated *TP*53 gene. Numerous tumor suppressors and oncogenes were found in the gene signature of these two subtypes suggesting that these subtypes contain invasive tumors. It is noteworthy that these subtypes are different in terms of crucial protein complexes and gene signature. Moreover, the *Perlious* tumors have a lower probability of survival.

The RF classification algorithm was used for supervised classification to detect subtypes for new breast cancer patients. Also, 16 critical genes were identified using RF that can be used for detecting breast cancer subtypes of new tumors. Consequently, the MSDEC subtypes obtained from somatic mutations were clinically meaningful and provide an informative insight into molecular subtype diagnosis and suggesting efficient clues for cancer treatment.

For future research, we intend to use the proposed method to detect subtypes of other cancers, such as glioblastoma. Moreover, we aim to use other data such as gene expression and methylation features of tumors for finding more appropriate subtypes. Furthermore, we propose to examine the importance of each data in detecting cancer subtypes.

## Data Availability Statement

Publicly available datasets were analyzed in this study. This data can be found at: https://github.com/nrohani/MolecularSubtypes.

## Author Contributions

NR and CE conceived the analysis. NR implemented the method, calculated the results, and wrote the manuscript. CE helped to improve the paper. Both authors have read and approved the final manuscript.

## Conflict of Interest

The authors declare that the research was conducted in the absence of any commercial or financial relationships that could be construed as a potential conflict of interest.

## Footnotes

^1^Genetics Home References, CDH1 gene, URL: https://ghr.nlm.nih.gov/gene/CDH1#normalfunction (accessed March 7, 2020).

^2^Genetics Home References, CBFB gene, URL: https://ghr.nlm.nih.gov/gene/CBFB#synonyms (accessed March 7, 2020).

^3^Genetics Home References, TBX3 gene, (Yarosh et al., 2008).

^4^Targeted Cancer Care, BRAF gene, URL: http://targetedcancercare.massgeneral.org/My-Trial-Guide/Diseases/Breast-Cancer/BRAF.aspx (accessed March 7, 2020).

^5^My Cancer Genome, GNAS gene, URL: https://www.mycancergenome.org/content/gene/gnas (accessed March 7, 2020).

^6^Genetics Home References, KRAS gene, URL: https://ghr.nlm.nih.gov/gene/KRAS (accessed March 7, 2020).

^7^Cancer Genetics Web, NSD3 gene, URL: http://www.cancerindex.org/geneweb/WHSC1L1.htm (accessed March 7, 2020).

^8^Genetics Home References, PTEN gene, URL: https://ghr.nlm.nih.gov/gene/PTEN#conditions (accessed March 7, 2020).

^9^My Cancer Genome, KDM5C gene, URL: https://www.mycancergenome.org/content/gene/kdm5c (accessed March 7, 2020).
