# Supplementary material for: Classifying Breast Cancer Molecular Subtypes by Using Deep Clustering Approach
Source: Front Genet. 2020 Nov 25;11:553587. doi: 10.3389/fgene.2020.553587 (PMC7723873; doi:10.3389/fgene.2020.553587)
Supplement: Supplementary file 1 [file Data_Sheet_1.PDF]

Supplementary Material

1 SUPPLEMENTARY TABLES AND FIGURES

1.1 Figures

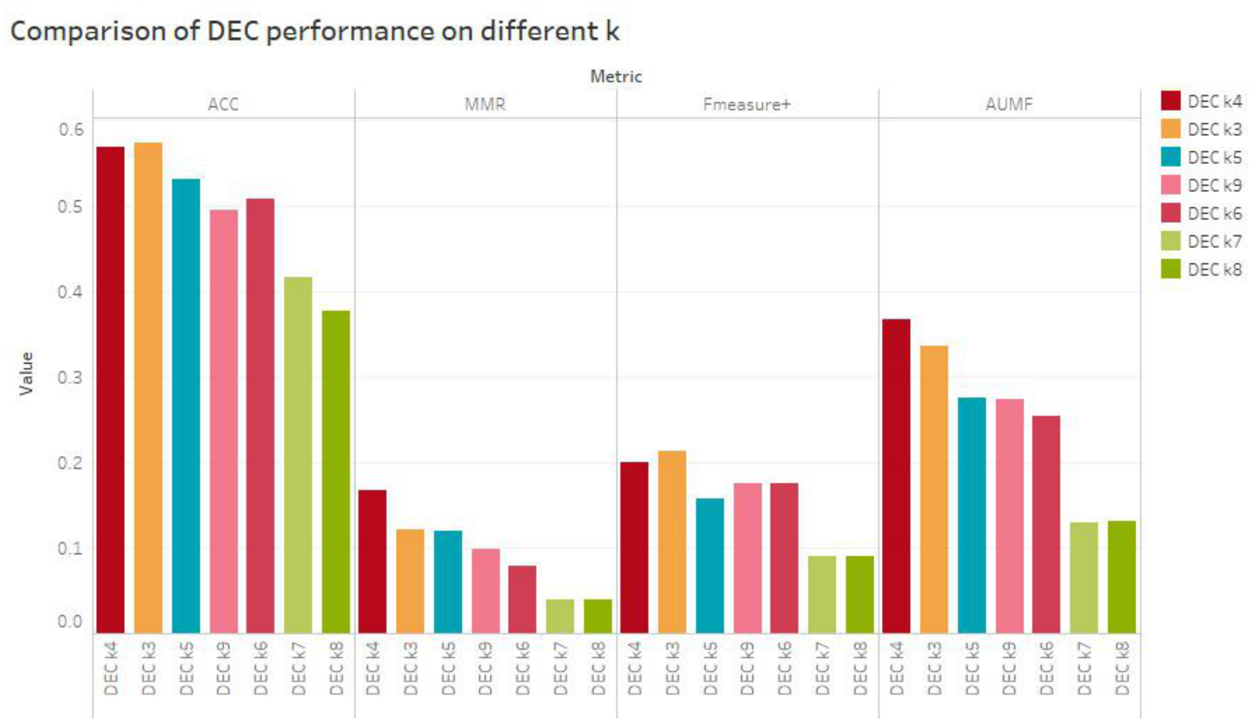

Figure S1: DEC performance on different ks.

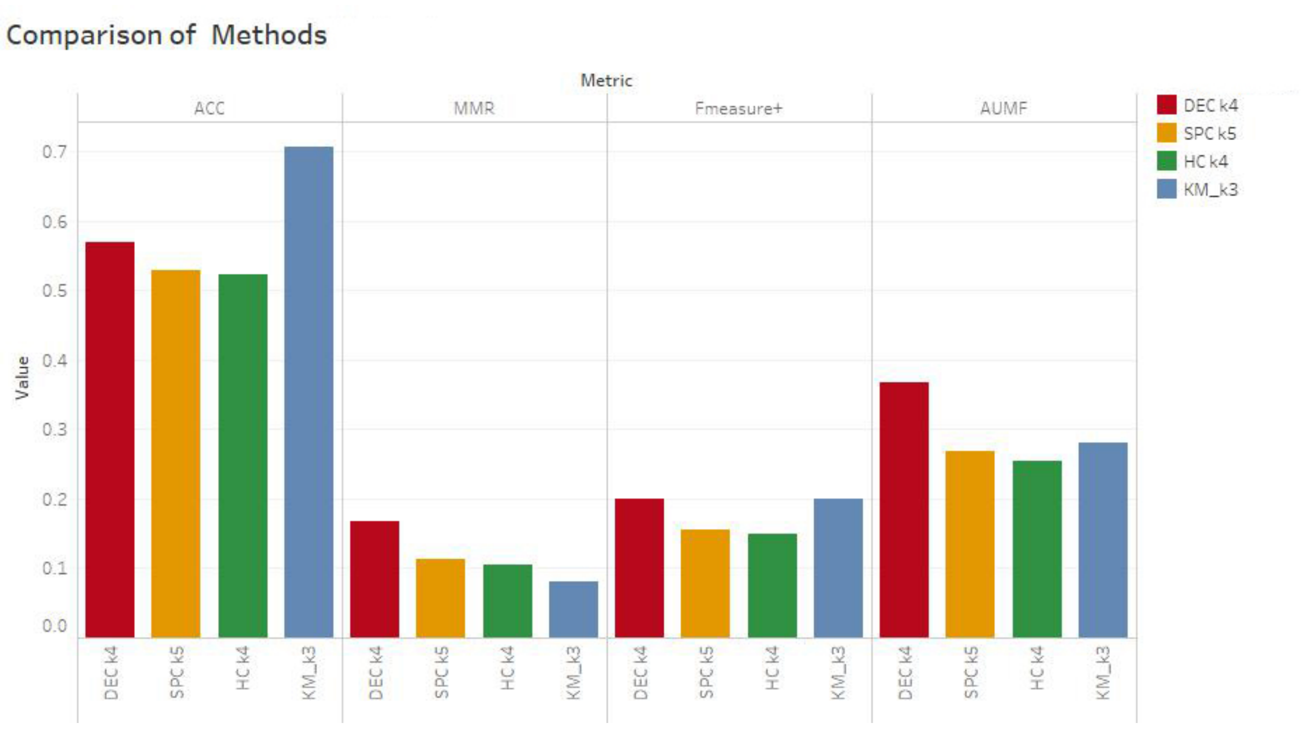

Figure S2: Comparison of the DEC results with other clustering methods.

Primary

GENE	
CDH1	7.37e-05
TBX3	1.98e-04
MAP3K1	2.78e-04
CTCF	4.42e-04
CBFB	1.32e-03
PIK3CA	1.77e-03
MED23	2.05e-03
FBXO11	7.70e-03
GATA3	9.91e-03
COL4A1	1.17e-02
GPS2	1.36e-02
MAP4K1	1.76e-02
RASGRF2	1.76e-02
TAOK1	1.76e-02
TSC1	2.09e-02
CDKN1B	2.35e-02
TBL1XR1	2.35e-02
RUNX1	2.72e-02
EFTUD2	3.39e-02
EXT2	3.39e-02
ITGA11	3.39e-02
PRKCQ	3.39e-02
PTPRJ	3.39e-02
PIK3C2G	3.94e-02
TNC	3.94e-02
CD22	4.42e-02
DKK4	4.42e-02
PRLR	4.42e-02
TRIP10	4.42e-02
COL5A3	4.82e-02

Pvalue

7.37e-05 4.82e-02

Figure S3: Top gene signature of $Primary$ subtype.

Progressive

GENE	Pvalue
TP53	3.30e-13
MYC	6.12e-08
GNAS	1.03e-06
ERBB2	1.58e-06
CACNA1F	2.34e-06
FGFR3	4.23e-06
AKT3	8.51e-06
BRAF	1.71e-05
RB1	2.33e-05
MPL	2.52e-05
AKT1	5.43e-05
CACNA1S	6.99e-05
FANCA	6.99e-05
CCNE1	9.07e-05
CCR7	1.13e-04
CDKN2A	1.23e-04
MACF1	1.47e-04
EGFR	1.65e-04
JAK1	2.38e-04
SRSF2	3.12e-04
LAMB4	3.59e-04
STK11	3.59e-04
BRCA1	4.10e-04
ANAPC5	4.70e-04
SPOP	5.79e-04
CARD11	6.55e-04
TLN1	6.55e-04
ARID4B	9.39e-04
COL4A6	9.39e-04
SETDB1	9.39e-04
KIT	1.07e-03
BRCA2	1.15e-03
NCOA3	1.23e-03
CLASP2	1.31e-03
RARB	1.31e-03
BCL2	1.69e-03
EZH2	1.84e-03
LAMB1	1.84e-03
MYB	1.84e-03
IDH2	2.20e-03
SKP2	2.52e-03
DDX3X	2.84e-03
GATA2	2.84e-03
NCF2	2.84e-03
ROCK2	2.84e-03
STAT4	2.84e-03
TNFAIP3	2.84e-03
TNKS1BP1	2.84e-03
IGF1R	3.22e-03
ARID1B	3.41e-03

Pvalue: 3.30e-13 – 3.41e-03

Figure S4: Top gene signature of $Progressive$ subtype.

Proliferous

GENE	
MYC	4.09e-07
IGF1R	9.16e-07
TP53	1.26e-06
KRAS	1.93e-05
ERBB2	4.68e-05
WHSC1L1	6.31e-05
SPOP	1.17e-04
IDH2	2.37e-04
KMT2C	3.51e-04
SMARCA1	3.51e-04
CCND1	5.16e-04
IKBKB	6.10e-04
AR	8.02e-04
BTK	9.64e-04
PSIP1	9.64e-04
CCNE1	1.56e-03
PIK3C2B	1.78e-03
MEN1	2.13e-03
TBL1X	2.13e-03
HCFC1	2.63e-03
RB1	3.54e-03
F8	4.60e-03
EGFR	5.46e-03
ATM	5.87e-03
TERT	6.15e-03
NOTCH1	6.70e-03
VWF	6.70e-03
PTEN	7.65e-03
CARD11	8.38e-03
FOXL2	8.38e-03
STAG2	8.38e-03
BCL9	1.07e-02
CYP4F11	1.07e-02
EML4	1.07e-02
NCOR2	1.07e-02
NUP107	1.07e-02
RTN4	1.07e-02
SPRED1	1.07e-02
DLG1	1.41e-02
ERBB4	1.41e-02
LAMA2	1.41e-02
MYH11	1.41e-02
UBC	1.41e-02
COL6A3	1.58e-02
DNMT1	1.62e-02
CBL	1.81e-02
CHEK2	1.81e-02
COL2A1	1.81e-02
CTNNA3	1.81e-02
MYLK2	1.81e-02

Pvalue

4.09e-07 1.81e-02

Figure S5: Top gene signature of $Proliferous$ subtype.

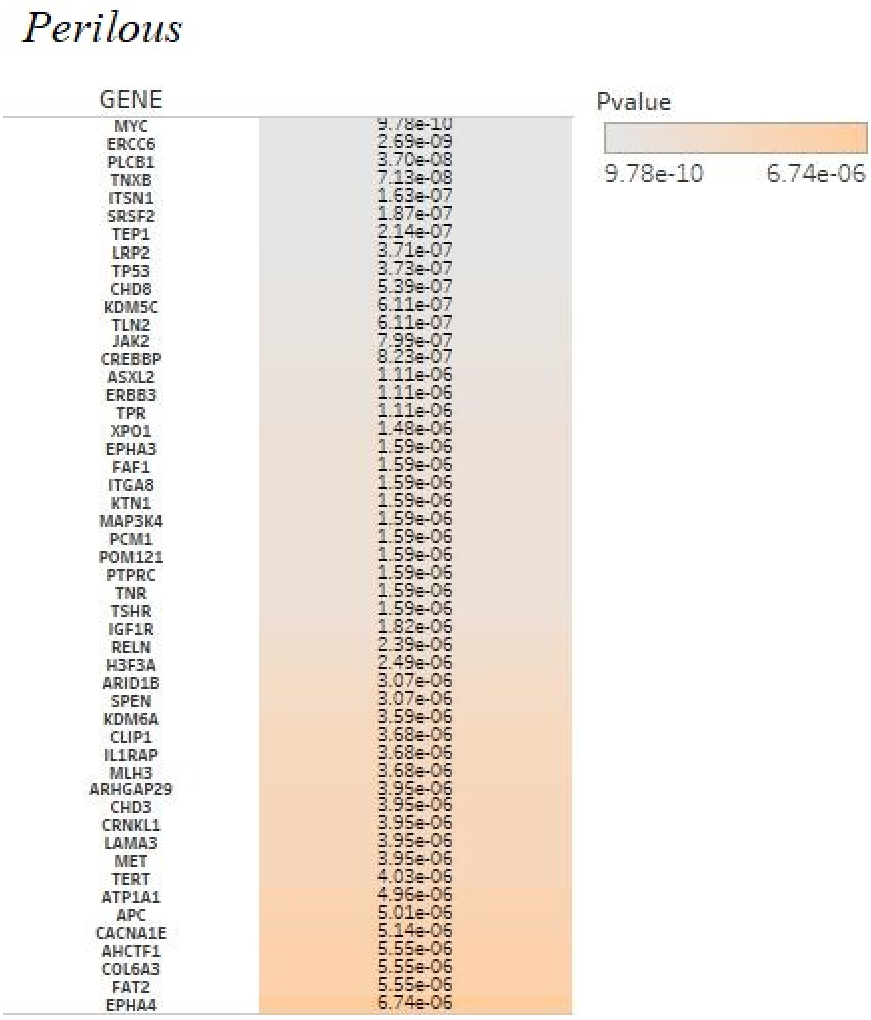

Perilous

GENE	Pvalue
MYC	9.78e-10
ERCC6	2.69e-09
PLCB1	3.70e-08
TNXB	7.13e-08
ITSN1	1.63e-07
SRSF2	1.87e-07
TEP1	2.14e-07
LRP2	3.71e-07
TP53	3.73e-07
CHD8	5.39e-07
KDM5C	6.11e-07
TLN2	6.11e-07
JAK2	7.99e-07
CREBBP	8.23e-07
ASXL2	1.11e-06
ERBB3	1.11e-06
TPR	1.11e-06
XPO1	1.48e-06
EPHA3	1.59e-06
FAF1	1.59e-06
ITGA8	1.59e-06
KTN1	1.59e-06
MAP3K4	1.59e-06
PCM1	1.59e-06
POM121	1.59e-06
PTPRC	1.59e-06
TNR	1.59e-06
TSHR	1.59e-06
IGF1R	1.82e-06
RELN	2.39e-06
H3F3A	2.49e-06
ARID1B	3.07e-06
SPEN	3.07e-06
KDM6A	3.59e-06
CLIP1	3.68e-06
IL1RAP	3.68e-06
MLH3	3.68e-06
ARHGAP29	3.95e-06
CHD3	3.95e-06
CRNKL1	3.95e-06
LAMA3	3.95e-06
MET	3.95e-06
TERT	4.03e-06
ATP1A1	4.96e-06
APC	5.01e-06
CACNA1E	5.14e-06
AHCTF1	5.55e-06
COL6A3	5.55e-06
FAT2	5.55e-06
EPHA4	6.74e-06

Figure S6: Top gene signature of *Perilous* subtype.

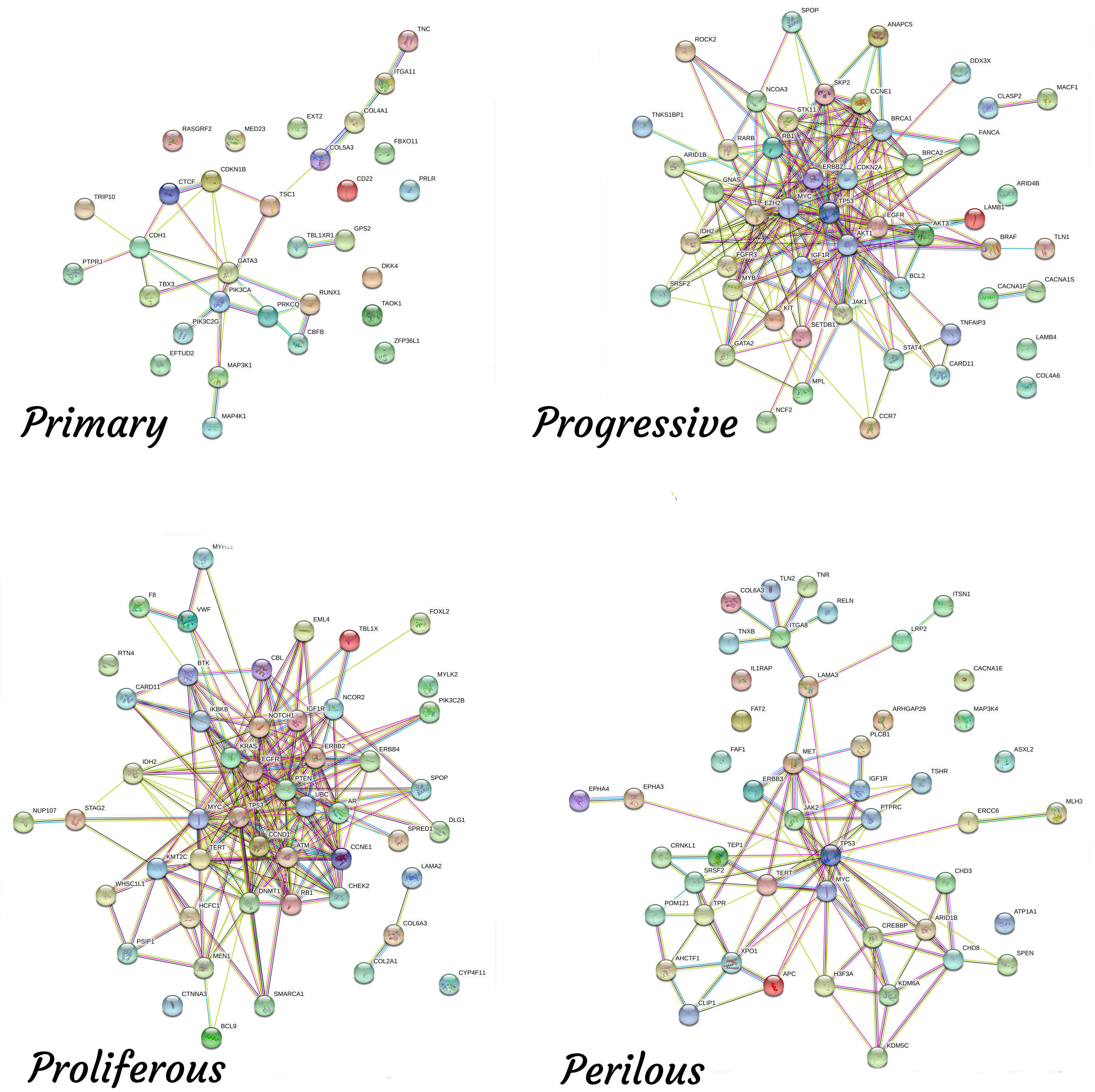

Figure S7: PPI network for each subtype.

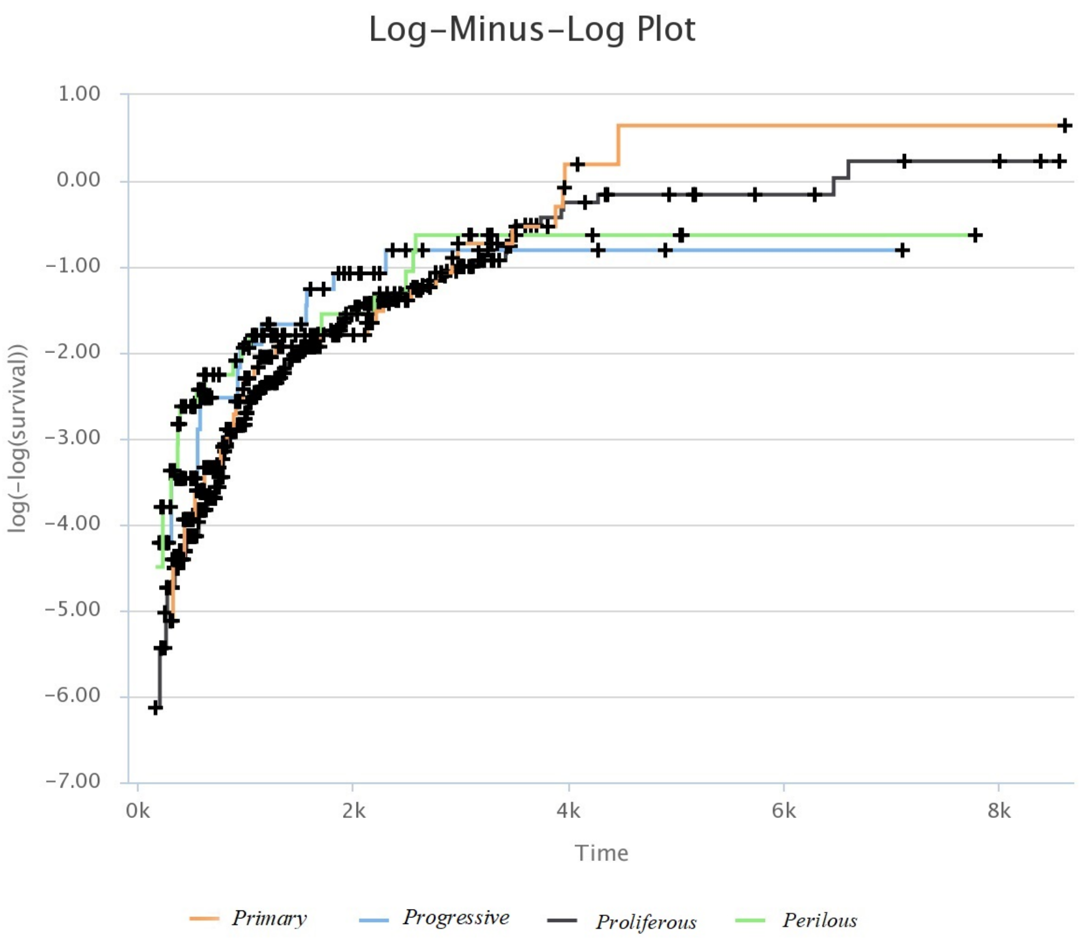

Figure S8: Cox hazard survival diagram.

Breast Carcinoma Estrogen Receptor Status

	Negative	Positive
Primary	49	125
Progressive	35	40
Proliferous	53	412
Perilous	44	49

Figure S9: Contingency table of ER status relation with discovered subtypes ($p - value < 2.2e - 16$ by Chi-square test and $p - value = 1e - 06$ by Fisher's exact test).

Breast Carcinoma Progesterone Receptor Status

	Negative	Positive
Primary	64	109
Progressive	47	29
Proliferous	93	369
Perilous	54	39

Figure S10: Contingency table of PR status relation with discovered subtypes ($p - value < 2.2e - 16$ by the Chi-square test and $p - value = 1e - 06$ by the Fisher's exact test.)

	Lab Proc Her2 Neu Immunohistochemistry Receptor Status		
	Equivocal	Negative	Positive
Primary	24	88	44
Progressive	5	40	22
Proliferous	91	265	44
Perilous	15	44	23

Figure S11: Contingency table of HER2 status relation with discovered subtypes ($p - value = 1.445e - 07$ by the chi-square test and $p - value = 1e - 06$ by the Fisher's exact test).

	TP53 Mutation Status	
	p53mut	p53wt
Primary	88	94
Progressive	43	34
Proliferous	92	396
Perilous	61	37

Figure S12: Contingency table of TP53 status relation with discovered subtypes ($p - value < 2.2e-$ in both the Chi-squared test and the Fisher's exact test).

	Histological Type						
	Infiltrating Ductal Carcinoma	Infiltrating Lobular Carcinoma	Medullary Carcinoma	Metaplastic Carcinoma	Mixed Histology	Mucinous Carcinoma	Other, specify
Primary	147	23	3	1	1	2	5
Progressive	69	7					1
Proliferous	331	103		2	20	10	22
Perilous	80	8	2		2		4

Figure S13: Contingency table of histopathological subtypes relation with discovered subtypes ($p - value = 0.0001615$ by the chi-square test and $p - value = 5.4e - 05$ by the Fisher's exact test).

	MSDEC Subtypes			
	Primary	*Progressive*	*Proliferous*	*Perilous*
Luminal A	72	18	338	15
Luminal B	52	16	99	28
Basal-like	35	30	46	38
Her2	23	18	16	17

Figure S14: Contingency table of discovered subtypes relation with PAM50 subtypes ($p - value < 2.2E - 16$ by the Chi-square test and $p - value = 1E - 06$ by the Fisher's exact test).

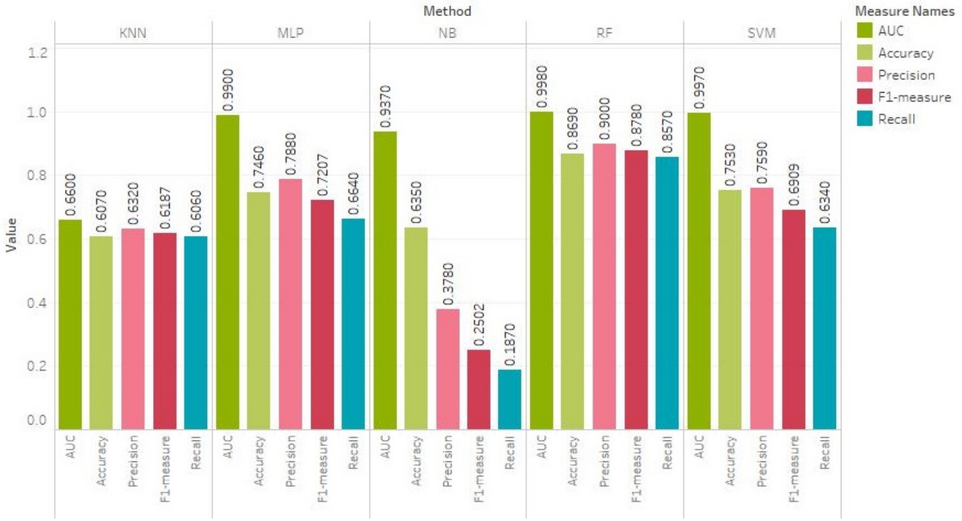

Figure S15: Comparison of random forest method with other classification methods.

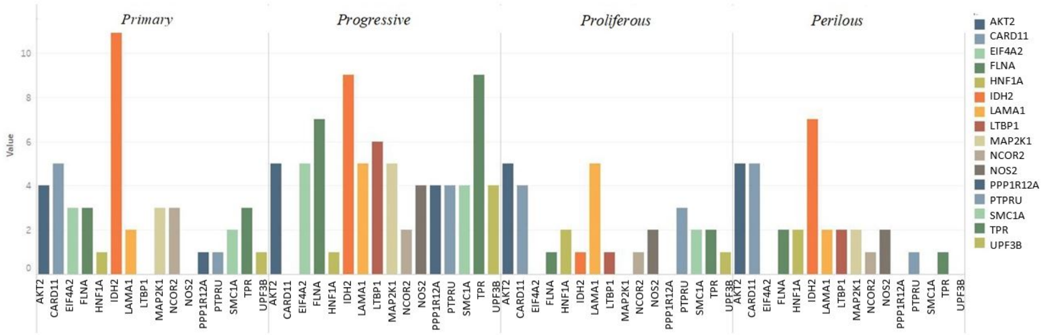

Figure S16: Mutational frequency of 16 important genes for classification of tumors in each subtype.

	cytokines and growth factors	transcription factors	homeodomain proteins	cell differentiation markers	protein kinases	translocated cancer genes	oncogenes	tumor suppressors
tumor suppressors	0	1	0	1	0	0	0	4
oncogenes	0	1	0	0	0	2	3	
translocated cancer genes	0	1	0	0	0	2		
protein kinases	0	0	0	0	4			
cell differentiation markers	0	0	0	3				
homeodomain proteins	0	0	0					
transcription factors	0	6						
cytokines and growth factors	1							

Figure S17: Family of gene signature in $Primary$ subtype.

	cytokines and growth factors	transcription factors	homeodomain proteins	cell differentiation markers	protein kinases	translocated cancer genes	oncogenes	tumor suppressors
tumor suppressors	0	2	0	0	1	0	0	8
oncogenes	0	3	0	4	6	5	15	
translocated cancer genes	0	2	0	1	2	5		
protein kinases	0	0	0	4	10			
cell differentiation markers	0	0	0	6				
homeodomain proteins	0	0	0					
transcription factors	0	11						
cytokines and growth factors	0							

Figure S18: Family of gene signature in $Progressive$ subtype.

	cytokines and growth factors	transcription factors	homeodomain proteins	cell differentiation markers	protein kinases	translocated cancer genes	oncogenes	tumor suppressors
tumor suppressors	0	2	0	0	2	0	0	6
oncogenes	0	3	0	1	2	8	14	
translocated cancer genes	0	2	0	0	0	9		
protein kinases	0	0	0	2	9			
cell differentiation markers	0	0	0	2				
homeodomain proteins	0	0	0					
transcription factors	0	8						
cytokines and growth factors	0							

Figure S19: Family of gene signature in $Proliferous$ subtype.

	cytokines and growth factors	transcription factors	homeodomain proteins	cell differentiation markers	protein kinases	translocated cancer genes	oncogenes	tumor suppressors
tumor suppressors	0	2	0	0	0	0	0	4
oncogenes	0	2	0	0	1	6	8	
translocated cancer genes	0	2	0	0	0	6		
protein kinases	0	0	0	1	6			
cell differentiation markers	0	0	0	2				
homeodomain proteins	0	0	0					
transcription factors	0	8						
cytokines and growth factors	0							

Figure S20: Family of gene signature in $Perilous$ subtype.

1.2 Tables

$Complex\ Name$	$Proteins$
$NCOR\ complex$	NCOR1, HDAC3, GPS2, TBL1XR1, TBL1X, CORO2A
$SMRT\ complex$	TBL1X, TBL1XR1, GPS2, HDAC3, NCOR2
$Kaiso - NCOR\ complex$	CORO2A, TRIM33, TBL1X, TBL1XR1, GPS2, HDAC3, KIF11, KDM4A, NCOR1, ZBTB33
$NCOR - HDAC3\ complex$	TBL1X, TBL1XR1, GPS2, HDAC3, NCOR1
$Polycystin - 1 - E - cadherin - beta - catenin\ complex$	CDH1,CTNNB1, PKD1
$p27 - cyclinE - CDK2\ complex$	CDK2, CDKN1B, CCNE1
$p27 - cyclinE - Cdk2\ -\ Ubiquitin\ E3\ ligase\ complex$	SKP1A, SKP2, CUL1, CKS1B, RBX1,CCNE1, CDKN1B, CDK2
$Polycystin - 1 - E - cadherin - beta - catenin - Flotillin - 2\ complex$	CDH1,CTNNB1, FLOT2, PKD1
$Polycystin - 1\ multiprotein\ complex$	ACTN1, CDH1, SRC, JUP, VCL, CTNNB1, PXN, BCAR1, PKD1, PTK2, TLN1

Table S1. Protein complexes of $Primary$ subtype.

$Complex\ Name$	$Proteins$
$BRCC\ complex$	BRE, BRCA1, BRCA2, RAD51, BRCC3
$MSH26 - BLM - p53 - RAD51\ complex$	BLM, TP53, MSH2, MSH6, RAD51
$BRCA1 - BARD1 - BACH1 - DNA\ damage\ complex\ II$	BARD1, BRCA1, RBBP8, RAD50, TOPBP1, MRE11A, NBN, BACH1
$BRCA1 - BARD1 - BACH1 - DNA\ damage\ complex\ I$	BARD1, BRCA1, MLH1, MSH6, TOPBP1, BACH1
$BRCA1 - BARD1 - BACH1 - DNA\ damage\ complex\ III$	BARD1, BRCA1, RBBP8, RAD50, TOPBP1, MRE11A, NBN, BACH1
$BRCA1A\ complex$	FAM175A, UIMC1, BARD1, BRCA1
$BRCA1C\ complex$	UIMC1, BARD1, BRCA1, RBBP8
$BRCA1B\ complex$	BARD1, BRCA1, BACH1
$BRCA1 - CTIP - ZBRK1\ repressor\ complex$	BRCA1, RBBP8, ZNF350
$BARD1 - BRCA1 - CSTF64\ complex$	BARD1, BRCA1, CSTF2
$BRAF53 - BRCA2\ complex$	BRCA2, HDAC1, HDAC2, KDM1A, PHF21A, RCOR1, HMG20B
$BRCA1 - BARD1 - POLR2A\ complex$	BARD1, BRCA1, POLR2A
$BRCA1 - CtIP - CtBP\ complex$	BRCA1, CTBP1, RBBP8
$BRCA1 - LMO4 - CTIP\ complex$	BRCA1, RBBP8, LMO4
$BRCA1 - BARD1 - UbcH7c\ complex$	BARD1, BRCA1, UBE2L3

Table S2. Protein complexes of $Progressive$ subtype.

$Complex\ Name$	$Proteins$
$Paf\ complex$	PAF1, CTR9, LEO1, WDR61
$p27 - cyclinE - CDK2\ complex$	CDK2, CDKN1B, CCNE1
$MDC1 - MRN - ATM - FANCD2\ complex$	MRE11A, FANCD2, MDC1, NBN, ATM, RAD50
$MDC1 - H2AFX - TP53BP1\ complex$	H2AFX, MDC1, TP53BP1
$MDC1 - p53BP1 - SMC1\ complex$	MDC1, SMC1A, TP53BP1
$SH3KBP1 - CBLB - EGFR\ complex$	CBLB, EGFR, SH3KBP1
$E2F4 - p130\ complex$	RBL2, E2F4
$CIN85 - CBL\ complex$	CBL, SH3KBP1
$MLL - HCF\ complex$	SERPIND1, KMT2A, HCFC1 , MEN1, MEN1, KDM6B, ASH2L, WDR5
$RAB5 - EEA1\ complex$	EEA1, RAB5A
$EGFR - CBL - GRB2\ complex$	CBL, EGFR, GRB2
$NCOR\ complex$	NCOR1, HDAC3, GPS2, TBL1XR1, TBL1X, CORO2A
$SMRT\ complex$	TBL1X, TBL1XR1, GPS2, HDAC3, NCOR2
$SMRT\ core\ complex$	TBL1X, HDAC3, NCOR2

Table S3. Protein complexes of $Proliferous$ subtype.

$Complex\ Name$	$Proteins$
$Anaphase - promoting\ complex$	ANAPC1, ANAPC10, ANAPC2, ANAPC4, ANAPC5, ANAPC7, CDC16,CDC23,CDC27
$SHARP - CtBP\ complex$	CTBP1,CTBP2,SPEN
$MDC1 - H2AFX - TP53BP1\ complex$	H2AFX,MDC1,TP53BP1
$MDC1 - p53BP1 - SMC1\ complex$	MDC1, SMC1A, TP53BP1
$JAK2 - PAFR - TYK2\ complex$	PTAFR, JAK2, TYK2
$Hes1 - Tle1\ complex$	Hes1, Tle1
$PLCB1 - PARD3 - PARD6A\ complex$	PLCB1, PARD3, PARD6A
$RSmad\ complex$	ARID1B, CREBBP, TRIM33, SMAD2,SMAD3 ,NCOA3,SMAD4,SMARCC1,SMARCC2, SMARCA4 (BAF190A)
$PTIP - HMT\ complex$	KMT2B, KMT2C, KDM6A, NCOA6, PAXIP1, PAGR1, DPY30, KDM6B, ASH2L, WDR5
$UTX - MLL2/3\ complex$	KMT2C, KMT2D, KDM6A, N4BP2, NCOA6, PAXIP1, PROSER1, KDM6B, PPP6R3, ASH2L, WDR5, ZNF281
$ITGAV - ITGB3 - LAMA4\ complex$	ITGAV,ITGB3,LAMA4
$HES1\ promoter\ corepressor\ complex$	CREBBP, CDK7, POLR2A, EP300, RBPJ, SUPT6H
$ESR1 - MDM4\ complex$	ESR1, MDM4

Table S4. Protein complexes of $Perilous$ subtype.
